# Bone marrow-derived mesenchymal stem cells mitigate chronic colitis and enteric neuropathy via anti-inflammatory and anti-oxidative mechanisms

**DOI:** 10.1038/s41598-024-57070-6

**Published:** 2024-03-20

**Authors:** Rhian Stavely, Ainsley M. Robinson, Sarah Fraser, Rhiannon T. Filippone, Vanesa Stojanovska, Rajaraman Eri, Vasso Apostolopoulos, Samy Sakkal, Kulmira Nurgali

**Affiliations:** 1 Institute for Health and Sport, Victoria University, Melbourne, VIC Australia; 2 Department of Pediatric Surgery, Massachusetts General Hospital, Harvard Medical School, Boston, MA 02114 USA; 3 The Ritchie Centre, Hudson Institute of Medical Research, Clayton, VIC Australia; 4 School of Science, STEM College, RMIT University, Melbourne, VIC Australia; 5 Immunology Program, Australian Institute of Musculoskeletal Science (AIMSS), Melbourne, VIC Australia; 6 Department of Medicine Western Health, Faculty of Medicine, Dentistry and Health Sciences, The University of Melbourne, Melbourne, VIC Australia; 7 Regenerative Medicine and Stem Cells Program, Australian Institute of Musculoskeletal Science (AIMSS), Melbourne, VIC Australia; 8 Enteric Neuropathy Lab, Western Centre for Health, Research and Education, St Albans, VIC 3021 Australia

**Keywords:** Mesenchymal stem cell, Colitis, Enteric nervous system, Oxidative stress, Neuroinflammation, BM-MSC, Gastroenterology, Medical research, Neurology

## Abstract

Current treatments for inflammatory bowel disease (IBD) are often inadequate due to limited efficacy and toxicity, leading to surgical resection in refractory cases. IBD’s broad and complex pathogenesis involving the immune system, enteric nervous system, microbiome, and oxidative stress requires more effective therapeutic strategies. In this study, we investigated the therapeutic potential of bone marrow-derived mesenchymal stem cell (BM-MSC) treatments in spontaneous chronic colitis using the *Winnie* mouse model which closely replicates the presentation and inflammatory profile of ulcerative colitis. The 14-day BM-MSC treatment regimen reduced the severity of colitis, leading to the attenuation of diarrheal symptoms and recovery in body mass. Morphological and histological abnormalities in the colon were also alleviated. Transcriptomic analysis demonstrated that BM-MSC treatment led to alterations in gene expression profiles primarily downregulating genes related to inflammation, including pro-inflammatory cytokines, chemokines and other biomarkers of inflammation. Further evaluation of immune cell populations using immunohistochemistry revealed a reduction in leukocyte infiltration upon BM-MSC treatment. Notably, enteric neuronal gene signatures were the most impacted by BM-MSC treatment, which correlated with the restoration of neuronal density in the myenteric ganglia. Moreover, BM-MSCs exhibited neuroprotective effects against oxidative stress-induced neuronal loss through antioxidant mechanisms, including the reduction of mitochondrial-derived superoxide and attenuation of oxidative stress-induced HMGB1 translocation, potentially relying on MSC-derived SOD1. These findings suggest that BM-MSCs hold promise as a therapeutic intervention to mitigate chronic colitis by exerting anti-inflammatory effects and protecting the enteric nervous system from oxidative stress-induced damage.

## Introduction

Current treatments for inflammatory bowel disease (IBD), consisting of the pathologies ulcerative colitis (UC) and Crohn’s disease (CD), are frequently ineffective in halting the disease progression or can only be used for short durations due to their toxicity. Consequently, surgical resection of the affected bowel is required in up to 80% of CD and 30% of UC refractory cases^1,2^. The inadequacy of current treatments may be reflective of a lack of understanding of the disease pathogenesis, which is considered to include interlinked dysfunction in the immune system, enteric nervous system (ENS), microbiome, and oxidative stress.

The gastrointestinal (GI) tract is innervated by the ENS, a component of the autonomic nervous system. Essential for normal intestinal function, the ENS regulates the GI tract effector systems, including smooth muscles, secretory glands, and blood-lymphatic vasculature. Structurally the ENS consists of two major plexuses of enteric neurons and glia. The outer myenteric plexus is located between smooth muscles in the bowel and controls contraction and relaxation patterns. The inner submucosal plexus is situated between the muscular and epithelial layers and regulates mucosal functions such as permeability, secretion and absorption, leukocyte migration and blood flow^3,4^. It is evident that inflammation-induced ENS impairment has profound ramifications on pivotal physiological functions, such as intestinal motility^5–11^. Additionally, nicotine intake and the presence of immune cells in proximity to the enteric plexuses (plexitis) of the ENS are reliable indicators of inflammatory relapse after surgery, suggestive of a neuroimmune component in the pathophysiology of severe intestinal inflammation^12–14^. As a result, the ENS has surfaced as a novel and promising therapeutic target for IBD^15–17^.

Mesenchymal stem cells (MSCs) have been proposed as an alternative treatment for IBD and their therapeutic value has been observed in patients who are refractory to conventional treatments^18,19^. Despite these promising results, achieving sustained remission is a significant hurdle given that repeated MSC administration is impractical due to the cost associated with the adoptive transfer of high-dose MSCs (in some cases as high as 10^9^ cells) (reviewed in Tian et al.^19^). Therefore, the mechanism of action of MSC therapy requires elucidation to either enhance the efficacy of MSCs or develop other therapeutics that target similar pathways. MSCs are capable of simultaneously acting on several therapeutic pathways and have demonstrated anti-inflammatory, trophic, neuroprotective and anti-oxidative properties in various disease models^20,21^. MSC immunomodulatory properties have been a key focus in the treatment of intestinal inflammation, predominately investigated in models of murine acute chemically-induced colitis^22^. Recent clinical data have not supported certain immunomodulatory mechanisms of MSC therapy in IBD, which were reported in experimental models of chemically-induced colitis in rodents, such as the induction of FOXP3 + regulatory T lymphocytes (Treg)^23^. This may be due to the acute nature of inflammation in chemically-induced colitis, which does not mimic the chronic inflammatory signaling milieu of IBD.

To gain insight into the mechanistic properties of MSCs in chronic colitis, we utilized the *Winnie* (*Muc2*^wnn^) mouse model of spontaneous chronic colitis with an inflammatory profile highly representative of UC to characterize the effects of bone marrow-derived MSC (BM-MSC) therapies^24,25^. Here, we provide evidence using transcriptional analysis that BM-MSC treatments are anti-inflammatory in experimental spontaneous chronic colitis with many genes of key pertinence to UC. Unbiased predictions in cell type changes via transcriptomic analysis reveal that enteric neuronal signatures are the most affected by BM-MSC treatment of chronic inflammation. Further investigation suggests that the neuroprotective properties of MSC treatments in colitis could prevent necrotic-like cell death by targeting cytokines such as high mobility group box 1 protein (HMGB1) whose levels are elevated in the faeces of colitis patients. HMGB1 plays a crucial role in the regulation of inflammation, acting as both a pro-inflammatory cytokine and a danger signaling molecule. It is released by necrotic cells and has been shown to contribute to neuropathies in experimental colitis^26^. Here we demonstrate that by targeting molecules such as HMGB1 we can unlock the therapeutic potential of MSCs through antioxidant and anti-inflammatory mechanisms.

## Results

### BM-MSC treatments reduce the severity of spontaneous chronic colitis

**Figure 1:**
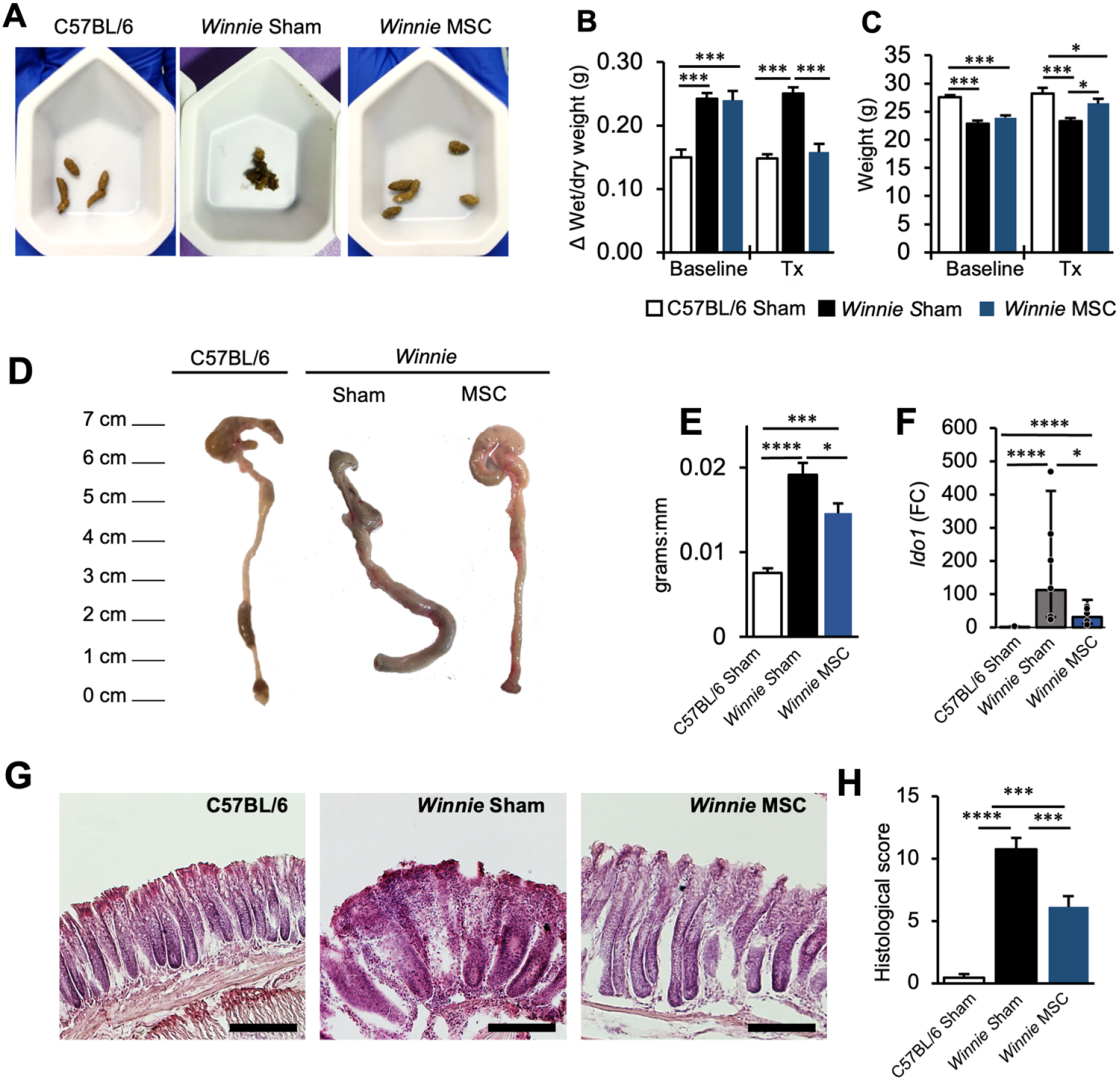
Mesenchymal stem cells (MSCs) supress the severity of spontaneous chronic colitis. (**A**) Representative images of fecal pellets collected at 14 days post-MSC treatment. (**B**) Delta wet weight/dry weight of fecal pellets and (**C**) body weight (grams) immediately before treatment (BT) and at 14 days post-treatment (Tx). (**D**) Gross morphology of colons and (**E**) quantification of the colonic weight:length ratio from C57BL/6 (n = 5), sham-treated *Winnie*, and MSC-treated *Winnie* mice (n = 7/group). (**F**) Expression of *Ido1* in the distal colon (n = 6 mice/group). Data presented as fold change with upper and lower 95% confidence limits. (**G**) Representative images of haematoxylin and eosin staining in the distal colon of C57BL/6, sham-treated *Winnie*, and MSC-treated *Winnie* mice (scale bar = 50 µm) and (**H**) quantification of histological scores C57BL/6 (n = 7), sham-treated *Winnie* (n = 8), and MSC-treated *Winnie* mice (n = 7). Data are presented as mean ± SEM unless otherwise stated and analyzed by one-way ANOVA and the Holm–Sidak method post hoc *P < 0.05; ***P < 0.001 and ****P < 0.0001 in all datasets.

To determine the therapeutic value of MSCs in spontaneous chronic colitis *Winnie* mice were treated with BM-MSCs via enema, an application method that has been demonstrated previously to be effective in acute models of chemically-induced colitis with minimal off-target sequestration in abdominal cavities and filtering organs^27–34^. The diarrheal sequela in *Winnie* mice was attenuated by the 14-day BM-MSC treatment regime (Fig. 1A, B) and mice began to recover in body mass which had been stunted by chronic colitis (Fig. 1C). Colons collected from sham-treated *Winnie* mice were morphologically distinct from C57BL/6 mice colons with an observable thickening in colon diameter, soft content and dark color which was less pronounced in *Winnie* mice treated with BM-MSCs (Fig. 1D). There was no difference in colon length of C57BL/6, sham-treated and BM-MSC-treated *Winnie* mice (Fig. S1A). Conversely, colon weight (Fig. S1B) and weight:length ratios (Fig. 1E) were significantly increased in *Winnie*-sham compared to C57BL/6 mice which were reduced by BM-MSCs treatments. The molecular biomarker of murine colitis, *Ido1*, was reduced 3.7-fold by BM-MSC treatments in the distal colon of *Winnie* mice (Fig. 1F). Likewise, treatment with BM-MSCs reduced the gross morphological damage to the *Winnie* mouse colon including changes to crypt architecture, crypt length and presence of abscesses, tissue damage, loss of goblet cells and leukocyte infiltration^24^ (Fig. 1G, H).

### Anti-inflammatory pathways and gene expression after BM-MSC treatments in chronic colitis

**Figure 2:**
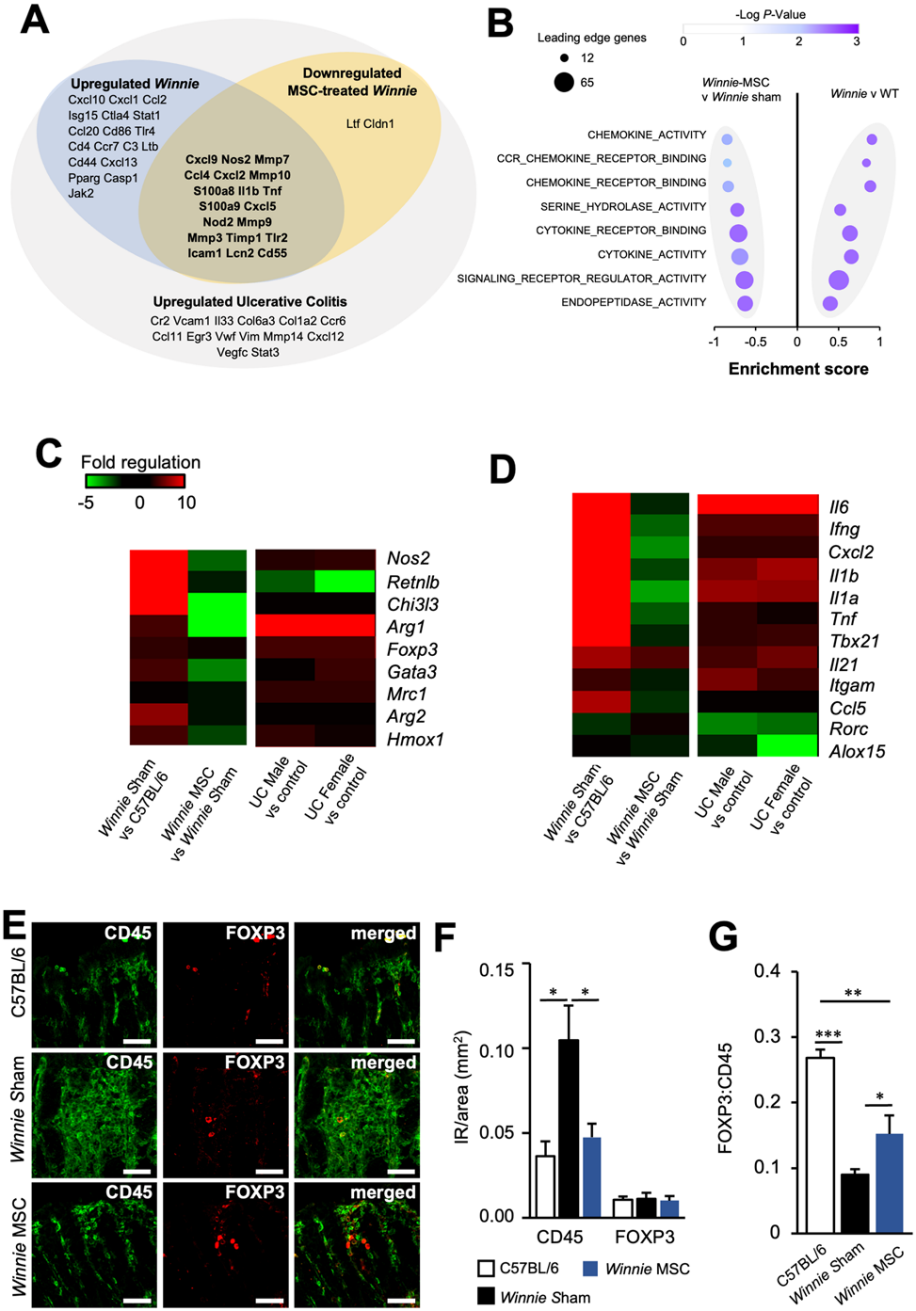
Mesenchymal stem cell treatments function via alternate anti-inflammatory pathways in spontaneous chronic colitis. (**A**) Venn-diagram presentation of differentially expressed genes (DEGs) downregulated by MSCs in *Winnie* mice compared to transcriptomic markers of ulcerative colitis (UC)^35^. (**B**) Top enriched terms from the Gene ontology (GO—molecular function) identified by GSEA between sham-treated *Winnie* and wildtype C57Bl/6 or MSC-treated *Winnie* and sham-treated *Winnie* mice. (**C**, **D**) Gene expression of selected factors previously determined to be upregulated (**C**) or downregulated (**D**) by MSC treatments in models of chemically-induced colitis. Data presented as fold regulation between the distal colons of MSC-treated (*Winnie* MSC) and sham-treated *Winnie* mice (*Winnie* Sham), *Winnie* Sham and C57BL/6 mice, as well as UC or Crohn’s disease colon compared to uninflamed controls. (**E**) Representative images of immunohistochemistry for CD45 (left column), FOXP3 (middle column) and merged images (right column) in cross sections of the distal colon (scale bar = 50 µm). (**F**) Quantification of the immunoreactive (IR) area of CD45^+^ and CD45^+^FOXP3^+^ cells in C57BL/6 (n = 4), sham-treated *Winnie* (n = 7), and MSC-treated *Winnie* mice (n = 7). Data presented as mm^2^ per image field and are the mean ± SEM, analyzed by one-way ANOVA and the Holm-Sidak method post hoc. *P < 0.05. (**G**) Quantification of the ratio of the CD45^+^FOXP3^+^ to CD45^+^ IR area in C57BL/6 (n = 4), sham-treated *Winnie* (n = 7), and MSC-treated *Winnie* mice (n = 7). Data are presented as mean ± SEM and analyzed by one-way ANOVA and the Holm–Sidak method post hoc. *P < 0.05; **P < 0.01; ***P < 0.001.

To evaluate the effects of BM-MSC treatments on the inflammatory profile of colitis, the transcriptome was assessed by RNA-Seq of the distal colon. Deferentially expressed genes (DEG) were determined revealing 1839 DEGs between sham-treated *Winnie* and C57BL/6 mice, and 228 DEGs between sham-treated and BM-MSC-treated *Winnie* mice. The gene expression profile of *Winnie* mice was compared to genes upregulated in UC patients identified by Holgerson et al.^35^ using transcriptomic profiling of literature-defined IBD-related genes (Fig. 2A). The *Winnie* mouse model was representative of UC with 37 of 53 UC genes upregulated (Chi-squared, *p* < 0.00001). Treatments with MSCs reduced the expression of 21 of these UC-associated genes, including the pro-inflammatory cytokines, *Il1b* and *Tnf,* Matrix metalloproteinases, *Mmp7*, *Mmp10*, *Mmp9* and *Mmp3,* ROS and nitrosylation genes, *Nos2*, *S100a8* and *S100a9*, pathogen sensing genes, *Nod2* and *Tlr2,* and the antimicrobial gene *Lcn2.* The chemokines *Cxcl9*, *Ccl4*, *Cxcl2* and *Cxcl5*, as well as *Icam1* which regulates leukocyte transmigration (Fig. 2A). Gene set enrichment analysis (GSEA) was performed on ranked gene expression between *Winnie* mice receiving sham or MSC treatments. Analysis utilizing the GO molecular function database revealed that MSCs were predominately associated with a decline in chemokines and cytokines (Fig. 2B, Tables S1–S2).

Factors previously associated with the anti-inflammatory activity of MSCs in animal models of chemically-induced colitis were identified by a survey of the literature (Table S3). Changes in the corresponding genes were assessed in *Winnie* mice treated with BM-MSCs and baseline changes were determined in *Winnie* mice and IBD patients compared to their uninflamed controls (Fig. 2C, D). Between models of chemically-induced colitis and *Winnie* mice, only half of the genes shared similar expression after MSC treatments. Similar to the reported effects of MSCs in chemically-induced colitis, BM-MSC treatments in *Winnie* mice downregulated the expression of *Il6, Ifng, Il1b, Il1a, Tnf, Cxcl2, Tbx21* and *Itgam*, which were upregulated in *Winnie* mice and IBD patients. Nevertheless, BM-MSC treatments downregulated *Nos2, Arg1, Gata3, Mrc1* and *Hmox1* which were upregulated in *Winnie* mice and IBD patients; but are reportedly also upregulated by MSCs in chemical models of colitis (Table S3). Genes representative of anti-inflammatory macrophage polarization (*Mrc1*, *Hmox1*, *Arg1* and *Chi3l3*) were either unchanged or downregulated by MSC treatments in contrast to some reports in acute chemically-induced colitis models (Fig. 2C, Table S3). Only *Foxp3* was upregulated by MSC treatments in *Winnie* mice and chemically-induced colitis, which is predominately expressed by Tregs, which are a FOXP3 expressing immune cell population.

Immune cell infiltration was assessed in cross sections of the distal colon using the pan-leukocyte marker CD45 (Fig. 2E). The average area of CD45-immunoreactivity (IR) was higher in sections from *Winnie* sham-treated compared to C57BL/6 mice. Treatment with BM-MSCs in *Winnie* mice reduced CD45-IR to levels comparable to C57BL/6 mice (Fig. 2F). No differences were observed between the total level of CD45^+^FOXP3^+^ leukocytes in the distal colon of C57BL/6, sham-treated and BM-MSC-treated *Winnie* mice (Fig. 2F). Nonetheless, the ratio of CD45^+^FOXP3^+^ to CD45^+^ leukocytes was decreased in *Winnie*-sham compared to C57BL/6 mice. A lower ratio was also observed in BM-MSC-treated *Winnie* mice to C57BL/6 mice; however the ratio of FOXP3^+^ leukocytes was elevated compared to sham-treated *Winnie* mice (Fig. 2G). These data indicated that a reduction in other leukocyte populations may be responsible for the increase in the FOXP3^+^ population. This is supported by gene expression data indicating that MSC treatments downregulated genes encoding several chemokines and enzymes responsible for extracellular matrix degradation with roles in the recruitment of leukocytes to the intestine during inflammation (Fig. 2A). Therefore, MSC treatments may suppress leukocyte recruitment by controlling extracellular matrix expression and the secretion of chemokines. Altogether, these data indicate that BM-MSC treatments reduce inflammation in chronic colitis, however these mechanisms may be different than those in the chemically-induced colitis models.

## MSCs protect the enteric nervous system from chronic colitis

**Figure 3:**
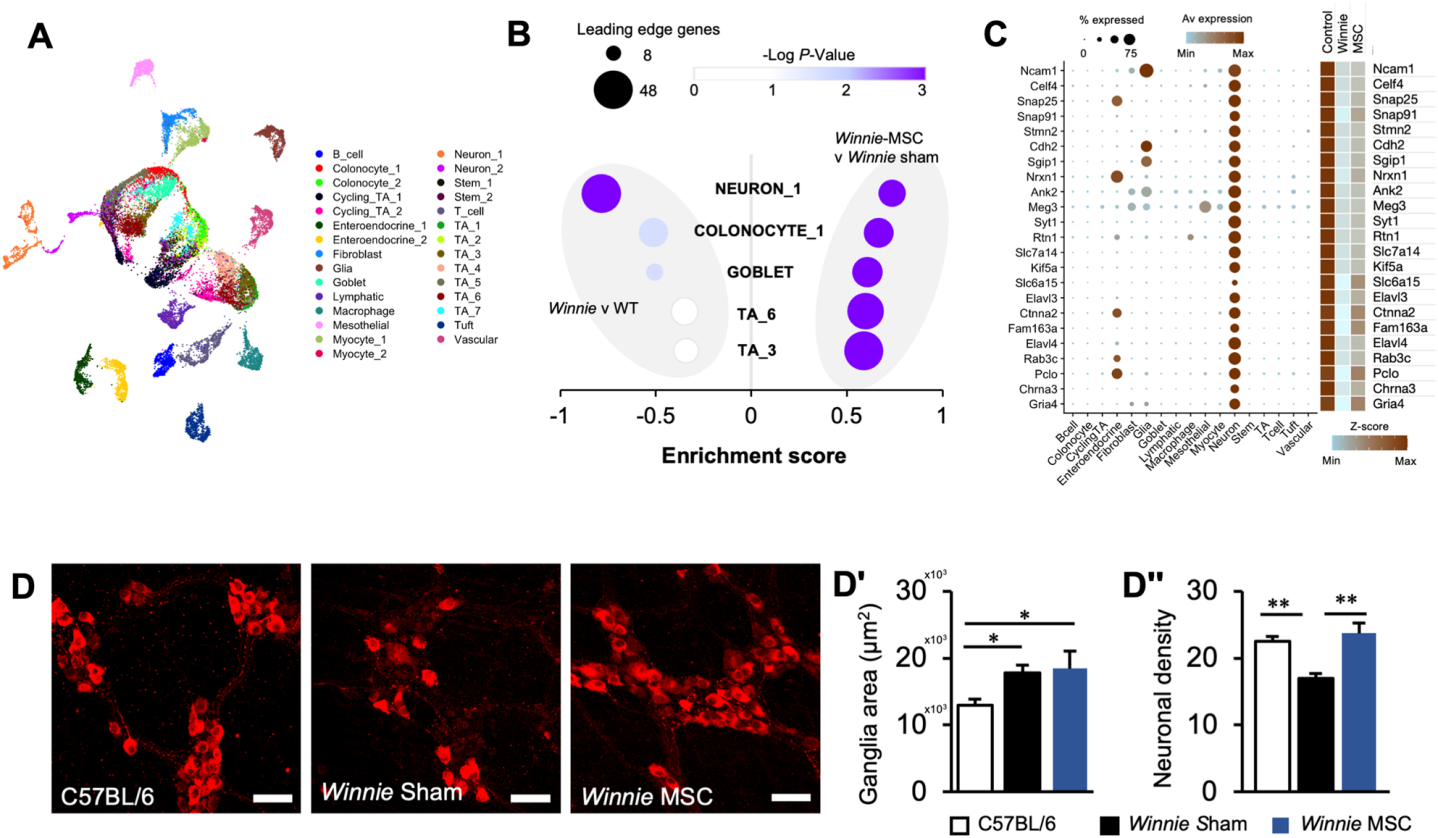
Mesenchymal stem cell treatments ameliorate colitis-associated enteric neuropathy. (**A**) UMAP projection of single cell RNA-Seq data from the C57BL/6 colon. (**B**) GSEA performed on genesets of colonic cell transcriptional profiles generated from Drokhlyansky et al. on ranked gene expression between *Winnie* and C57BL/6 mice or MSC-treated *Winnie* and sham-treated *Winnie* mice. (**C**) Dotplot visualization of the specificity of enteric neuron markers in colonic cells (left) and Heatmap representation of enteric neuronal genes in C57BL/6, sham-treated *Winnie* and MSC-treated *Winnie* mice (right). Data presented as Z-scores of gene expression (CPM). (**D**) Immunohistochemistry of the neuronal marker MAP-2 in longitudinal muscle-myenteric plexus (LMMP) wholemount preparations from the distal colon (scale bar = 50 µm). (**Dʹ**–**Dʹʹ**) Quantitative analysis of the average size of ganglionic units (**Dʹ**) and the myenteric neuronal density within the ganglia (**Dʹʹ**) in C57BL/6 (n = 6), sham-treated *Winnie* (n = 8) and MSC-treated *Winnie* mice (n = 5). All data are presented as mean ± SEM and analyzed by one-way ANOVA and the Holm-Sidak method post hoc. *P < 0.05; **P < 0.01; ***P < 0.001; ****P < 0.0001 in all datasets.

To explore the effects of BM-MSC therapies on cell populations during colitis we utilized colonic scRNA-seq data generated by Drokhlyansky, et al.^36^ from C57BL/6 mice to acquire unique gene signatures of colonic cell populations (Fig. 3A). These signatures were subsequently used as gene sets for GSEA of our bulk RNA-seq data in *Winnie* mice to predict changes to cell populations during chronic colitis and after BM-MSC treatment. Gene sets for immune cell populations including macrophages, T-cells and B-cells were enriched with a positive association (top-ranked, upregulated) in *Winnie* mice compared to controls, however no enrichment of leukocyte gene sets were observed in *Winnie* compared to those with BM-MSC treatment. Nonetheless, the strongest cell signature enriched after BM-MSC treatments came from a positive association with enteric neurons, which likewise was dramatically reduced between *Winnie* and control mice (Fig. 3B). We validated that the specificity of the main genes driving enrichment were highly expressed in neurons and observed that the same genes were expressed at their highest levels in controls and lowest in *Winnie* mice*,* with levels being partially restored by BM-MSC treatment (Fig. 3C).

To determine if alterations in the expression of ENS-associated genes could be elucidated by the size of the myenteric plexus we investigated MAP-2 expression in wholemount LMMP preparations of the distal colon. Despite gene expression data indicating neuron-associated genes were downregulated between sham-treated *Winnie* and C57BL/6 mice, the size of myenteric ganglia were larger in sham-treated *Winnie* compared to C57BL/6 mice (Fig. 3D). Likewise, the myenteric ganglia in colon preparations from C57BL/6 mice were smaller than BM-MSC-treated *Winnie* mice. There was no difference in the size of the ganglia between sham- and BM-MSC-treated *Winnie* mice. These data indicate that the size of myenteric ganglia did not account for an increase in the expression of ENS-specific genes after BM-MSC treatment (Fig. 3Dʹ). However, enumeration of the neuronal density within the ganglia revealed neuronal loss in sham-treated *Winnie* mice compared to C57BL/6 mice, which may support the reduced expression of neuronal genes. Treatment with BM-MSCs corrected the decline in neuronal density in *Winnie* mice to comparable levels observed in C57BL/6 mice (Fig. 3Dʹʹ). Similar results were observed in cross-sections of the distal colon (Fig. S2).

**Figure 4:**
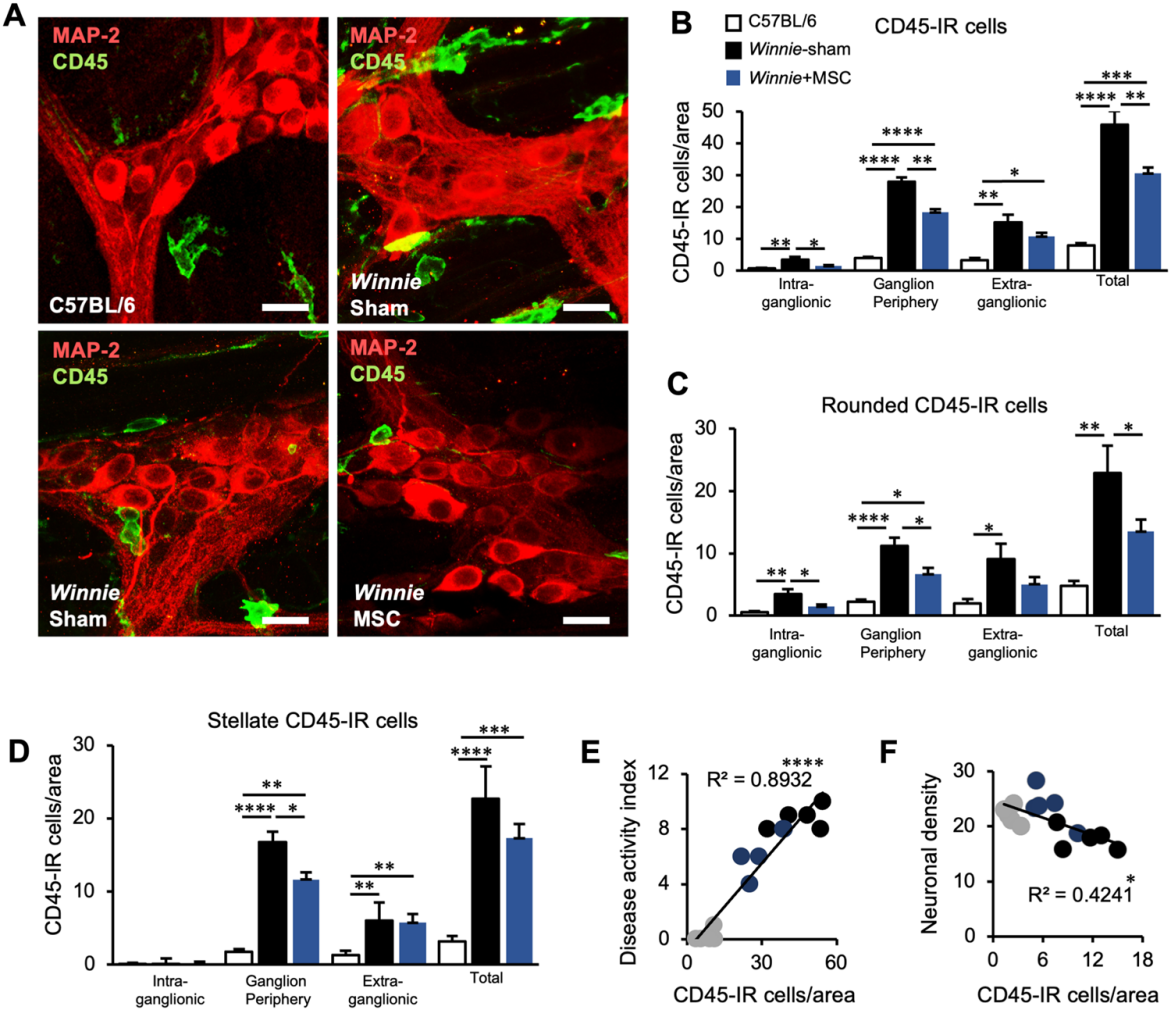
Plexitis is prevented by BM-MSC treatments. (**A**) Representative images of MAP-2-IR neurons and CD45-IR leukocytes in LMMP preparations from C57BL/6 mice, sham-treated *Winnie* mice (*Winnie* Sham) and MSC-treated *Winnie* mice (*Winnie* MSC). Top right image shows increased number of leukocytes around the myenteric ganglia in *Winnie* Sham, and bottom left image shows leukocyte infiltration into the myenteric ganglia in *Winnie* Sham (scale bar = 20 µm). (**B**) Quantification of CD-45-IR cells (leukocytes) in the myenteric plexus per area and categorised by location in proximity to the myenteric ganglia (intra-ganglionic, ganglion periphery and extra-ganglionic). (**C**, **D**) Leukocyte quantification after subdivision by rounded (**C**) and stellate-shaped (**D**) cellular morphology. n = 5 mice/group for all. (**E**, **F**) Correlations were observed between the total number of CD-45 IR cells/area and DAI scores (**E**), as well as the number of rounded CD-45-IR cells/area on the periphery of the ganglia and neuronal density (**F**).

Previously plexitis has been clinically associated with the severity of IBD^13,14,37,38^. Using the marker CD45, leukocytes were visualized in LMMP wholemount preparations (Fig. 4A). Leukocytes were observed in proximity to the myenteric ganglia using the pan-neuronal marker MAP-2. The majority of leukocytes lined the edges of the myenteric ganglia (ganglia periphery) in C57BL/6 and sham-treated *Winnie* mice. Many leukocytes were localized outside the myenteric ganglia (extra-ganglionic), however, a smaller number was also observed to infiltrate into the ganglia (intra-ganglionic) in direct contact with myenteric neurons. The total number of leukocytes quantified in LMMPs was significantly elevated in sham-treated *Winnie* mice compared to C57BL/6 mice (Fig. 4B). When leukocytes were quantified according to location, increased cell numbers were observed in all regions. In LMMP preparations from BM-MSC-treated *Winnie* mice, total leukocyte numbers remained elevated when compared to C57BL/6 mice. Nevertheless, compared to sham-treated *Winnie* mice, BM-MSC treatments reduced the total number of leukocytes, intra-ganglionic leukocytes and leukocytes on the ganglia periphery, but not in the extra-ganglionic region. Within the leukocytes at the level of the myenteric plexus, distinct morphological populations could be observed that resembled either a rounded or stellate structure, with the latter representing muscularis macrophages. Increased numbers of rounded leukocytes were observed in the intra-ganglionic area in sham-treated compared to BM-MSC-treated *Winnie* mice and C57BL/6 mice which contained similar levels (Fig. 4C). Furthermore, rounded leukocytes were increased on the periphery of the ganglia in sham-treated *Winnie* compared to C57BL/6 mice which were reduced by treatments with BM-MSC treatments. Region-specific differences were observed with BM-MSC treatments inducing a decrease in stellate-shaped leukocytes in *Winnie* mice on the periphery of the ganglia (Fig. 4D). A significant regression was found for the total number of leukocytes in proximity to the myenteric ganglia and DAI for colitis (Fig. 4E). This relationship appeared to be independent of leukocyte morphology or proximity to the ganglia with significant correlations observed in almost all permutations (Table S4). In addition, linear regression was used to determine the relationship between plexitis and neuropathy (Fig. 4F). The highest correlation was identified in a regression equation for the number of rounded leukocytes located on the periphery of the ganglia. Neuronal density was also negatively correlated with the number of rounded leukocytes in all regions or those that were intra-ganglionic (Table S5).

### Antioxidant mechanisms contribute to the neuroprotective effects of MSCs

**Figure 5:**
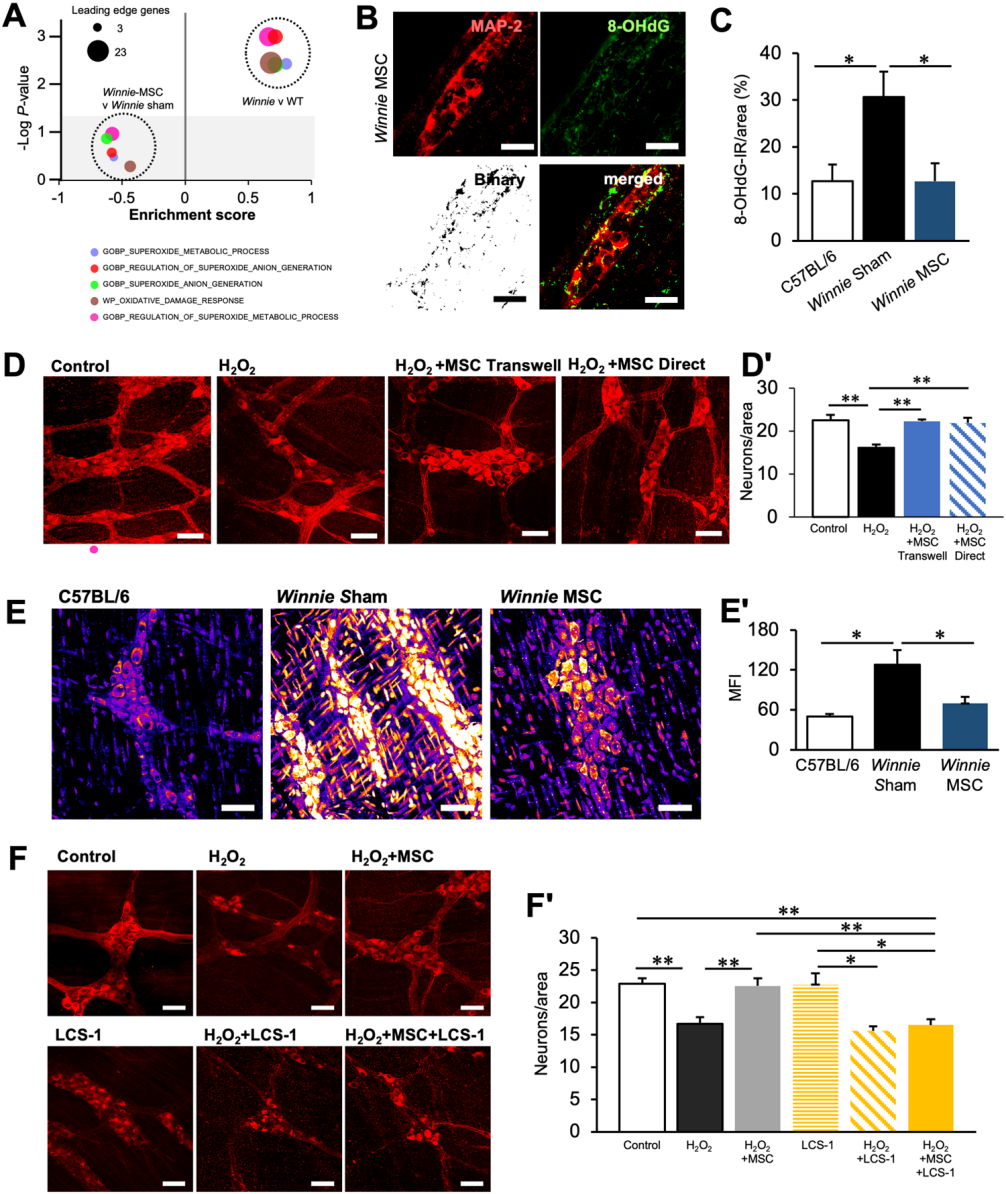
Mesenchymal stem cells directly protect enteric neurons from oxidative stress via anti-oxidant mechanisms in vivo and ex vivo. (**A**) GSEA using curated gene sets associated with oxidative stress and ranked lists of gene expression between *Winnie* and C57BL/6 mice or MSC-treated *Winnie* and sham-treated *Winnie* mice. The horizontal axis represents the enrichment score. Grey area represents data falling within a non-significant range (*P* > 0.05). (**B**) Immunofluorescence of 8-OHdG in neurons within the myenteric ganglia labelled with the neuronal marker MAP-2. Adducts of 8-OHdG were observed in binary images of 8-OHdG immunofluorescence. Scale bars = 20 µm. (**C**) Quantification of 8-OHdG adducts as a percentage area in the myenteric neurons in colon cross sections. C57BL/6: n = 6 mice, sham-treated *Winnie* and MSC-treated *Winnie*: n = 7 mice/group. (**D**) Neurons within the myenteric ganglia observed by immunofluorescence using MAP-2 in distal colon organotypic cultures exposed to 100 µM H_2_O_2_ and BM-MSCs. (**Dʹ**) Quantification of myenteric neuron density expressed as the number of neurons per ganglionated area. **P < 0.01; control: n = 6 independent samples, H_2_O_2_: n = 5 independent samples, H2O2 + MSC Transwell: n = 4 independent samples, H2O2 + MSC direct: n = 5 independent samples. (**E**) Mitochondria-derived superoxide (O_2_^−^) in the myenteric ganglia visualized by the fluorescent probe MitoSOX in LMMP wholemount preparations of the distal colon (scale bar = 50 µm). Presented images are pseudo-coloured (LUT: ‘heat’, ImageJ) for greater visual distinction. (**Eʹ**) Quantification of the mean fluorescence intensity of the myenteric ganglia in single channel 16-bit images of MitoSOX fluorescence. C57BL/6 and MSC-treated *Winnie*: n = 5 animals/group, sham-treated *Winnie*: n = 6 animals. (**F**) MAP-2-IR in distal colon organotypic cultures. Tissues were cultured for 24 h in standard culture medium and exposed to either H_2_O_2_, H_2_O_2_ with BM-MSCs, LCS-1 (SOD1 inhibitor), H_2_O_2_ with LCS-1 or H_2_O_2_ with LCS-1 and BM-MSCs (scale bar = 50 µm). (**Fʹ**) Quantification of myenteric neuron density expressed as the number of neurons per ganglionated area (control: n = 8 independent samples, H_2_O_2_: n = 9 independent samples, H_2_O_2_ + MSC n = 9 independent samples, LCS-1: n = 4 independent samples, H_2_O_2_ + LCS-1: n = 4 independent samples, H_2_O_2_ + MSC + LCS-1: n = 6 independent samples). All data are presented as mean ± SEM and analyzed by one-way ANOVA and the Holm–Sidak method post hoc. *P < 0.05; **P < 0.01; ***P < 0.001 and ****P < 0.0001 in all datasets.

Local oxidative stress is a primary driver of enteric neuropathy during intestinal inflammation^26,39^. To explore whether the presence of oxidative stress could be determined on the gene expression level in *Winnie* mice, gene sets associated with oxidative stress were curated from several databases to perform GSEA. These data indicated a positive association with terms related to oxidative stress in *Winnie* mice compared to controls, particularly several terms associated with the generation of superoxide (O_2_^−^) (Fig. 5A). However, no significant enrichment of oxidative stress-related terms were observed in *Winnie* mice treated with BM-MSCs (Fig. 5A). To validate the levels of oxidative stress in chronic inflammation, DNA/RNA adducts were assessed by immunohistochemistry in cross sections of the distal colon by labelling 8-OHdG. Localization of 8-OHdG to myenteric neurons was determined by measuring the area of 8-OHdG-IR adducts in myenteric neurons labelled by MAP-2 (Fig. 5B). In myenteric neurons, 8-OHdG-IR was elevated in sham-treated *Winnie* compared to C57BL/6 mice. In BM-MSC-treated *Winnie* mice, IR for 8-OHdG was restored to levels comparable to C57BL/6 mice (Fig. 5C).

As BM-MSCs did not alter the expression of genes associated with oxidative stress, we postulated that the lack of oxidative stress after treatment may be explained by a direct antioxidant effect rather than by acting via the expression of ROS-generating machinery and antioxidants in the recipient. This was explored by examining the effects of organotypic culture on neuronal loss in LMMPs from the distal colon of C57BL/6 mice. No differences were observed in the neuronal density of myenteric neurons between fresh fixed wholemount tissues (22.5 ± 0.7 neurons/area) and tissues that had been freshly peeled, subjected to organotypic culture for 24 h and then fixed (22.5 ± 1.2 neurons/area) (Fig. S3). Organotypic cultures were exposed to hyperoxic conditions to model oxidative stress in myenteric neurons (Fig. S4). A significant reduction in myenteric neuronal density was observed in tissues cultured in hyperoxia (5% CO_2_, 95% O_2_) compared to those cultured under atmospheric normoxia. BM-MSCs applied directly to organotypic cultures reduced the loss of myenteric neurons caused by hyperoxia. In addition, the neuroprotective effects of BM-MSCs in organotypic cultures were evaluated in oxidative stress using H_2_O_2_ (Fig. 5D). Application of H_2_O_2_ reduced neuronal density in organotypic cultures compared to tissues cultured for 24 h in standard culture medium (Fig. 5Dʹ). Neuronal loss induced by H_2_O_2_ was ameliorated by incubating organotypic cultures directly with BM-MSCs. Similar results were observed when organotypic cultures and BM-MSCs were separated by a semi-permeable transwell insert suggesting that MSCs reduce neuropathy in a paracrine fashion (Fig. 5Dʹ).

Mitochondria-derived O_2_^−^ was measured in the myenteric ganglia using the fluorescent dye MitoSOX. (Fig. 5E). In sham-treated *Winnie* mice, O_2_^−^ levels (mean fluorescence intensity) in the myenteric ganglia were more than twice the levels in C57BL/6 mice. Treatment with BM-MSCs reduced O_2_^.−^ levels in *Winnie* mice to levels comparable to controls (Fig. 5Eʹ). BM-MSCs have previously been shown to secrete SOD–Cu–Zn (SOD1), which has a primary role in the detoxification of mitochondria-derived O_2_^−^^40^. To examine the potential role of SOD1 in BM-MSC-mediated myenteric neuroprotection, the SOD1 inhibitor, LCS-1, was applied to organotypic cultures (Fig. 5F). Neuronal density was similar in organotypic preparations cultured in a standard medium and those treated with LCS-1 at a final concentration of 10 µM. Application of H_2_O_2_ with LCS-1 or without LCS-1 similarly reduced neuronal density compared to controls in standard culture medium and those with LCS-1 only (Fig. 5Fʹ). In cultures treated with H_2_O_2_, the application of BM-MSCs maintained neuronal density to levels observed in controls; however, when LCS-1 was added to these cultures, the neuroprotective effect of BM-MSCs was inhibited. All groups in these experiments contained 0.05% (v/v) DMSO either as a vehicle for LCS-1 or as a sham vehicle, which had no effect on neuronal density (Fig. S5).

## MSCs attenuate redox sensitive translocation of HMGB1 in myenteric neurons

**Figure 6:**
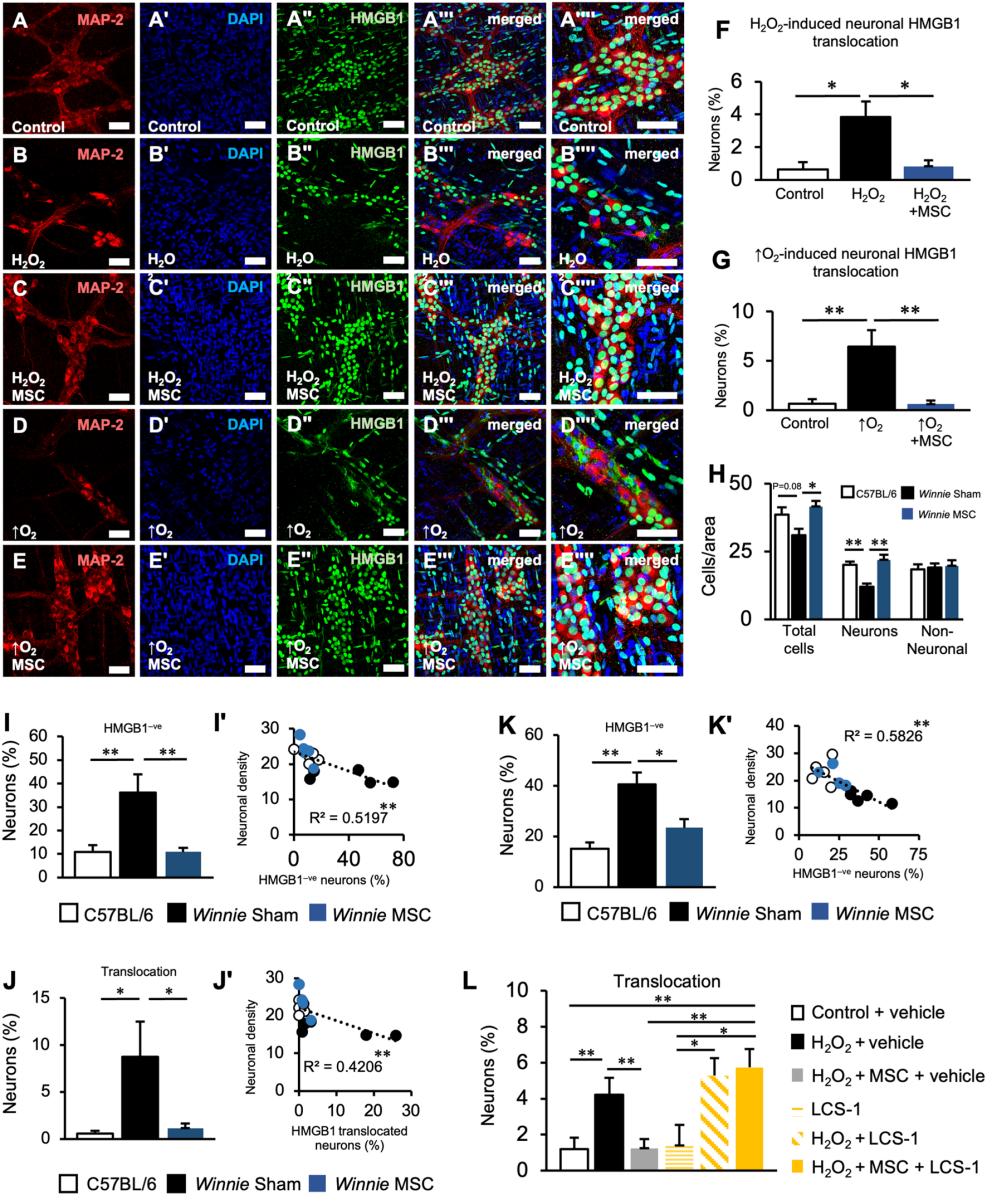
Mesenchymal stem cells prevent translocation of high-mobility group-box 1 associated with myenteric neuronal loss in colitis and oxidative stimuli. (**A**–**Eʹʹʹʹ**) HMGB1 translocation in organotypic cultures of myenteric neurons was observed by immunofluorescence of the MAP-2 (**A**–**E**), nuclear stain DAPI (**Aʹ**–**Eʹ**), HMGB1 (**Aʹʹ**–**Eʹʹ**), merged images (**Aʹʹʹ**–**Eʹʹʹ**) and merged magnified images (**Aʹʹʹʹ**–**Eʹʹʹʹ**). Tissues were cultured for 24 h in standard culture medium in 5% CO_2_ and ambient O_2_ conditions (**A**) and exposed to either 100 µM H_2_O_2_ (**B**), 100 µM H_2_O_2_ with 1 × 10^5^ BM-MSCs (**C**), hyperoxic (↑O_2_) conditions (5% CO_2_, 95% O_2_) (**D**) or hyperoxia with 1 × 10^5^ BM-MSCs (**E**) (scale bar = 50 µm). (**F**, **G**) Quantification of the percentage of neurons with HMGB1 translocated to the cytoplasm in co-cultures with BM-MSCs treated with 100 µM H_2_O_2_ (**F**) or under hyperoxic conditions (**G**). *P < 0.05; **P < 0.01; control: n = 7 independent samples, H_2_O_2_: n = 9 independent samples, H_2_O_2_ + MSC: n = 8 independent samples, ↑O_2_: n = 7 independent samples, ↑O_2_ + MSC: n = 5 independent samples. (**H**) Quantification of the total number of cells, neurons and non-neuronal cells with nuclear HMGB1 in the myenteric plexus of the distal colon of C57BL/6, sham-treated *Winnie* and MSC-treated *Winnie* mice expressed as cells per ganglionated area in LMMP. C57BL/6 and MSC-treated *Winnie*: n = 5 animals/group, sham-treated *Winnie*: n = 8 animals. (**I**, **J**) Percentage of neurons without HMGB1 in the nucleus (**I**) and HMGB1 translocated to the cytoplasm (**K**) in the LMMP. (**Iʹ**–**Jʹ**) Pearson correlation between neuronal density and the percentage of neurons without nuclear HMGB1 expression (**Iʹ**) or cytoplasmic HMGB1 translocation (**Jʹ**). C57BL/6 and MSC-treated *Winnie*: n = 5 animals/group, sham-treated *Winnie*: n = 8 animals. (**K**–**Kʹ**) Percentage of myenteric neurons without nuclear HMGB1 expression (**K**) and linear correlation between neuronal counts and the percentage of neurons without nuclear HMGB1 expression (**Kʹ**) in cross sections of the distal colon (n = 5 animals/group). (**L**) Quantification of the percentage of neurons with HMGB1 translocated to the cytoplasm in organotypic preparations exposed to either 100 µM H_2_O_2_, 100 µM H_2_O_2_ with 1 × 10^5^ BM-MSCs, 10 µM LCS-1 (SOD1 inhibitor), 100 µM H_2_O_2_ with 10 µM LCS-1 or 100 µM H_2_O_2_ with 10 µM LCS-1 and 1 × 10^5^ BM-MSCs. Control: n = 8 independent samples, H_2_O_2_: n = 9 independent samples, H_2_O_2_ + MSC n = 9 independent samples, LCS-1: n = 4 independent samples, H_2_O_2_ + LCS-1: n = 4 independent samples, H_2_O_2_ + MSC + LCS-1: n = 6 independent samples. All data are presented as mean ± SEM and analyzed by one-way ANOVA and the Holm–Sidak method post hoc. *P < 0.05; **P < 0.01; ***P < 0.001; ****P < 0.0001 in all datasets.

Cytoplasmic translocation and release of the redox-sensitive cytokine HMGB1 have been associated with oxidative stress during neuropathy of the CNS and ENS^26,41^. Considering that BM-MSCs were previously demonstrated to reduce oxidative stress in the myenteric neurons of *Winnie* mice, in vitro cultures were used to investigate the effect of BM-MSCs on oxidative stress-induced HMGB1 translocation. This was performed by exposing myenteric neurons to hyperoxia or H_2_O_2_ to induce oxidative stress and co-culturing them with BM-MSCs in semi-permeable transwell inserts (Fig. 6A–E). In H_2_O_2-_treated cultures, the application of BM-MSCs reduced the proportion of neurons with HMGB1 translocation into the cytoplasm to levels similarly observed in controls (Fig. 6F). Likewise, BM-MSCs inhibited HMGB1 translocation when cultured under hypoxic insult compared to cultures without BM-MSCs (Fig. 6G). These data indicated that BM-MSCs can inhibit neuronal HMGB1 translocation in oxidative conditions.

The number of neurons expressing HMGB1 in the nucleus was decreased in sham-treated *Winnie* compared to C57BL/6 mice (Fig. 6H). Loss of nuclear HMGB1 expression appeared to be specific to the neuronal population with no effects observed in non-neuronal cells within the myenteric ganglia in conditions of chronic inflammation (Fig. 6H). The total number of cells expressing HMGB1 in the nucleus was increased in BM-MSC-treated compared to sham-treated *Winnie* mice which was driven by an increase in nuclear HMGB1 expression in the neuronal population (Fig. 6H). The percentage of neurons without nuclear expression of HMGB1, or with HMGB1 translocation into the cytoplasm, was increased in sham-treated *Winnie* compared to C57BL/6 mice and was attenuated by BM-MSC treatments reduced the percentage of neurons without nuclear HMGB1 in *Winnie* mice (Fig. 6I, J). A negative correlation was observed between the proportion of neurons without nuclear HMGB1, or with HMGB1 translocation, and the neuronal density of the myenteric ganglia in vivo (Fig. 6Iʹ, Jʹ). These results were replicated in cross sections of the distal colon from a separate group of mice (Fig. 6K–Kʹ). Together, these data reflect the notion that the absence of nuclear HMGB1 and cytoplasmic translocation are indicative of a necrotic-like mechanism of cell death^42^.

Our previous experiments indicated that the ability for BM-MSCs to protect myenteric neurons from oxidative insult by H_2_O_2_ was mediated, at least in part, by SOD1 (Fig. 5F). Therefore, we assessed whether SOD1 was implemented in the ability of BM-MSCs to prevent HMGB1 translocation in myenteric neurons exposed to H_2_O_2_ (Fig. 6L). When SOD1 was inhibited in cultures with LCS-1, a similar degree of HMGB1 translocation was induced by H_2_O_2_ as vehicle controls compared to cultures with LCS-1 alone (Fig. 6L). No differences in HMGB1 translocation were observed between LCS-1 and vehicle controls, with or without exposure to H_2_O_2_. Inhibition of SOD1 negated the effects of BM-MSCs on H_2_O_2_-induced HMGB1 translocation; levels were elevated compared to BM-MSC co-cultures exposed to H_2_O_2_ and the controls, LCS-1 alone and vehicle alone. These data further support that BM-MSCs reduce HMGB1 translocation associated with neuropathy and identify the attenuation of oxidative insult as a mechanism of their neuroprotective action.

## Discussion

This study demonstrated that BM-MSCs can attenuate chronic colonic inflammation and the expression of inflammatory genes relevant to UC in the *Winnie* mouse model of spontaneous chronic colitis. BM-MSC treatments reduced the infiltration of leukocytes into the distal colon observed through histological and immunohistochemical analysis. The number of FOXP3^+^ leukocytes was unchanged by BM-MSC treatments; however, their proportion was increased due to a reduction in the total number of leukocytes. Analyses of the transcriptome revealed that BM-MSCs downregulated genes associated with UC and inflammatory processes and upregulated transcriptional signatures of enteric neurons. Oxidative stress has previously been shown to be responsible for enteric neuropathy during colitis^26,39^ and we identify that BM-MSCs could have a direct neuroprotective effect via antioxidant activity.

The therapeutic application of MSCs in acute experimental chemically-induced colitis has been extensively investigated. In these studies, MSCs attenuated rectal bleeding and inflammation-induced loss of body weight, improved stool consistency, and reduced histopathological severity of colitis after DSS or TNBS exposure in rats and mice^43–46^. In the *Winnie* model of spontaneous chronic colitis, BM-MSCs also alleviated these manifestations. In models of chemically-induced colitis, MSCs have been demonstrated to suppress leukocyte infiltration as indicated by decreased leukocyte counts in the mucosa and submucosa and reduced myeloperoxidase activity^31,47–49^. In our study, the number of leukocytes recruited to the distal colon was reduced to near control levels by BM-MSC treatment. Analysis of the transcriptome validated that the reduction in leukocyte numbers translated to a decrease in inflammatory chemokine and cytokine activity. Several transcriptional biomarkers of UC were suppressed including key pro-inflammatory cytokines *Il1b* and *Tnf* and neutrophil markers *S100a9, S100a8* and *Lcn2.* FOXP3 is a key transcription factor in the commitment to the Treg phenotype. Previous studies suggest that MSCs promote the polarization of Tregs^45,50^. The immunomodulatory effects of MSC treatment are associated with the number of Tregs in murine chemically-induced colitis^45,49–51^. Recent clinical trials were unable to replicate findings of FOXP3 + Treg polarization by MSCs in refractory luminal Crohn’s disease and data in UC are limited^23^. In our model of chronic colitis similar to UC, no differences were observed between the number of FOXP3 + leukocytes in the distal colon between C57BL/6 and *Winnie* mice administered sham or BM-MSC treatments. Nevertheless, the proportion of FOXP3 + leukocytes was increased by BM-MSC treatments. This may be rationalized by an overall decrease in leukocyte infiltration while the number of FOXP3 + leukocytes remains unchanged. Although FOXP3 is predominately expressed by Tregs, it can also be expressed by small subsets of different lymphocyte populations, therefore the lack of additional markers validating the Treg phenotype is a limitation in this experiment.

We performed an unbiased assessment of changes in cell populations via transcriptomic signatures. Interestingly, the strongest changes occurred in the enteric neuron signature which was downregulated in *Winnie* and upregulated after BM-MSC treatments. Recent studies have highlighted complex neuroimmune pathways in the intestine that suppress intestinal inflammation, therefore protecting the ENS may be pertinent to preventing both disharmony in neuroimmune pathways and the ENS-driven sequelae of IBD^9,52–54^. Nicotinic signaling in particular may be dysfunctional in UC as tobacco smoking reduces the incidence and severity of the disease and nicotine can promote remission independently^55,56^. Alterations to the ENS are reported in samples from IBD patients and animal models of colitis and may contribute to the progression and severity of the disease^6,7,11,57–61^. Gross structural degeneration is observed within the enteric ganglia in inflamed regions of UC^62,63^. Bernardini, et al.^57^ observed that the ganglionated area increases by 59% in patients with UC; when neuronal counts were normalized a 61% decrease in neuronal density was observed. Similar to UC patients, we observed reduced neuronal density in *Winnie* mice and furthermore this could be alleviated by BM-MSC treatments.

Plexitis is a highly prognostic marker of inflammatory relapse in IBD^12–14^. While the ability of MSCs to reduce leukocyte recruitment to the mucosa and submucosal layers has been reported extensively in chemically-induced colitis^31,47–49^, we observe that BM-MSC therapy reduced leukocyte numbers on the level of the myenteric ganglia which correlated well with reduced disease severity. The relationship between plexitis and disease activity appeared to be indiscriminate to the proximity of leukocytes to the ganglia and their morphology. In contrast, neuropathy appeared to be more dependent on the number of leukocytes with a rounded morphology located inside and on the edges of the myenteric ganglia. This may be consistent with recent reports of a macrophage-dependent degradation of the myenteric plexus barrier during colitis^64^, which could be a driver of enteric neuropathy.

Plexitis during intestinal inflammation has previously been associated with oxidative stress, particularly in the enteric neurons^26,39^. In IBD, oxidative stress has a bidirectional relationship with inflammation and is entrenched in the pathology of the disease^65,66^. The ENS is also considered to be susceptible to oxidative injury with enteric neuropathy linked to excessive ROS production and oxidative stress in animal models of chemotherapy, diabetes and physiological aging^67–70^. BM-MSCs decreased markers of oxidative stress and superoxide generation in myenteric neurons in Winnie mice. While genes associated with oxidative stress and free radical generation were upregulated in *Winnie* mice, we did not detect any clear reversal of these pathways via the sequenced mouse transcripts from the recipient after human BM-MSC treatment, which reflects endogenous expression profiles. MSCs themselves, however, exhibited neuroprotective properties in models of oxidative stress-induced neuropathy ex vivo in a paracrine manner, which offers evidence that MSCs can have direct antioxidant properties rather than acting via host tissues. BM-MSCs constitutively express and can secrete SOD1^40,71^ and MSCs from multiple sclerosis patients are deficient in SOD1 with a subsequent reduced neuroprotective capacity^72,73^. Considering that SOD1 is integral to detoxifying mitochondria-derived O_2_^−^, its role in BM-MSC-mediated neuroprotection was evaluated. Our results indicated that inhibition of SOD1 prevents BM-MSCs from rescuing neuropathy in a paracrine manner, which suggests that SOD1 could be a major part of their neuroprotective mechanism of action along with their ability to reduce inflammation and plexitis. It was also demonstrated that BM-MSCs prevent oxidative insult-induced translocation of HMGB1 from myenteric neurons. The relationship between plexitis and HMGB1 is not completely understood; however, redox-sensitive translocation of this pro-inflammatory cytokine has been shown in enteric neurons in response to oxidative stress^26^. The release of extracellular HMGB1 in the colon during colitis leads to activation of the NF-kB through a toll-like receptor—RAGE dependent manner, which in turn causes the upregulation of cytokines such as TNF-alpha^74^. These signaling pathways are associated with disrupting the function of tight junctions between cells, leading to increased permeability^75,76^, which may facilitate the infiltration of leukocytes into the enteric nerve plexus. Conversely, plexitis can also promote the release of HMGB1, perpetuating the inflammatory cycle in a feed-forward loop. Dysregulation of tight junction proteins may precede the development of colitis; thus, MSC administration in the early stages of the disease may attenuate disease severity, leading to better outcomes. However, in later stages that involve severe uncontrolled inflammation, such as refractory IBD, MSCs may act to prevent neural damage that sustains inflammation and has important ramifications on dysmotility.

In conclusion, this study elucidates the potential therapeutic mechanisms driving the efficacy of BM-MSCs in ameliorating chronic colonic inflammation and the associated inflammatory gene expression linked to UC. BM-MSC treatments exhibited notable reductions in leukocyte infiltration and transcriptomic profiling revealed the downregulatory effect on genes associated with inflammation while concurrently upregulating transcriptional signatures related to enteric neurons. Notably, BM-MSCs demonstrated the potential to directly counteract oxidative stress, a crucial contributor to enteric neuropathy during colitis, potentially via their direct antioxidant activity. These findings underscore the neuroprotective, anti-oxidative and anti-inflammatory potential of BM-MSC therapy, which could hold promise as an innovative approach for managing UC due to their ability to target the broad pathophysiological mechanisms driving chronic colitis.

## Materials and methods

### Animals

For in vitro organotypic studies, male C57BL/6 mice (14 weeks (wk) old) were obtained from the Animal Resource Centre (Perth, Western Australia, Australia). For in vivo experiments, male *Winnie* mice (14 weeks old) were obtained from Victoria University Werribee Animal Facility (Melbourne, Victoria, Australia) and compared to age matched male C57BL/6 controls. All mice had ad libitum access to food and water and were housed in a temperature-controlled environment with a 12 h day/night cycle. Mice were acclimatized for 1 week at the Western Centre for Health, Research and Education (Melbourne, Victoria, Australia). All mice were culled by cervical dislocation and the distal portion of the colon was collected for subsequent ex-vivo experiments. All animal experiments in this study complied with the ARRIVE guidelines 2.0^77^ and the guidelines of the Australian Code of Practice for the Care and Use of Animals for Scientific Purposes and were approved by the Victoria University Animal Experimentation Ethics Committee.

### Cell culture and passaging

Human BM-MSCs were obtained from Tulane University, New Orleans, LA, USA. BM-MSCs used in this study were previously extensively characterized for cell surface markers, differentiation potential, proliferation, colony formation, morphology, and adherence to plastic^27^ and conform to the guidelines set by the International Society for Cellular Therapy^78^. Cells were plated at a seeding density of 60 cells/cm^2^ and cultured at 37 °C in 5% CO_2_ and ambient air using expansion medium (α-MEM supplemented with 100 U/mL penicillin/streptomycin, 1% glutaMAX [Gibco®, Life Technologies, Melbourne, Australia, for all]) and 16.5% fetal bovine serum (FBS; mesenchymal stem cell-qualified, Gibco®, Life Technologies), which was replenished every 48–72 h for 10–14 days until the cells were 70–85% confluent. BM-MSCs used for animal treatments had a viability of over 95% and were used only at the fourth passage.

## MSC administration

BM-MSCs were administered to *Winnie* mice with chronic colitis by enema. A lubricated silicone catheter was inserted 3 cm proximal to the anus of mice anesthetized with 2% isoflurane. *Winnie* mice were treated with two doses of 4 × 10^6^ BM-MSCs in 100 µL of sterile PBS and subsequently received two replenishment doses of 2 × 10^6^ BM-MSCs in 100 µL sterile PBS as previously optimized in the *Winnie* model^79^. All treatments were administered 4 days apart. Sham-treated *Winnie* mice underwent the same procedure on the same days with an injection of 100 µL sterile PBS only (*Winnie*-sham). Mice were culled 24 h after the final treatment unless indicated otherwise.

### Evaluation of colitis

Clinical signs of colonic inflammation in *Winnie* mice include changes to colon weight and body weight, diarrhea, rectal prolapses, and rectal bleeding^24,25^. Colitis was confirmed by a disease activity index (DAI) as previously described^80^. To calculate fecal water content, pellets were collected from mice separated into individual sterile boxes for up to an hour, weighed, and dried in a fan-forced oven at 60 °C for 24 h to remove all moisture. Colitis was confirmed by a disease activity index (DAI) as previously described^80^. For histology, colon tissues were processed as previously described for staining with hematoxylin and eosin (H&E)^81^. Histological scores were evaluated for several parameters defined previously in the *Winnie* mouse model^24^.

### Immunohistochemistry and MitoSOX probe

Immunohistochemistry and MitoSOX staining were performed on wholemount preparations of the longitudinal muscle and myenteric plexus (LMMP) or cross sections of the distal colon as previously detailed^26^. Incubation with primary antibodies included: rat anti-CD45 (1:200) (BioLegend, San Diego, USA), rabbit anti-FOXP3 (1:500) (Abcam, Melbourne Australia), chicken anti-microtubule associated protein (MAP)-2 (1:5000) (Abcam, Melbourne Australia), rabbit anti-HMGB1 (1:2000; Abcam) or mouse anti-8-hydroxy-2'-deoxyguanosine (8-OHdG) (1:200; Abcam) with 2% normal donkey serum (NDS), overnight at 4 °C. For 8-OHdG immunolabelling, an additional blocking step for 1 h was performed prior to using goat unconjugated affinity purified F(ab) fragment anti-mouse IgG (H + L) antibody (1:100; Abcam, Melbourne, Australia). Secondary antibody labelling included: Alexa Fluor 488 donkey anti-rat, Alexa Fluor 594 or 647 donkey anti-rabbit, Alexa Fluor 594 donkey anti-chicken, Alexa Fluor 488 donkey anti-mouse (all 1:500; Jackson Immunoresearch, West Grove, USA) with 2% NDS for 1 h at room temperature. The production of O_2_^−^ in the myenteric plexus was assessed using MitoSOX Red (1:1000); Molecular Probes®, Thermofisher, Melbourne, Australia), a fluorogenic indicator of O_2_^−^ derived from the mitochondria^26^.

### Imaging and analysis

Immunoreactivity (IR) for CD-45, FOXP3, MAP-2 and HMGB1 or MitoSOX staining in cross sections or wholemounts were visualized using an Eclipse Ti confocal laser scanning system (Nikon, Tokyo, Japan). Identical image acquisition settings for each antibody labelling experiment were retained between all samples. Images were visualized using Image J v1.50b open source software (National Institute of Health, Bethesda, USA)^82,83^ with the Image J ND2 Reader plugin and were converted into maximum intensity projections in 16-bit.TIFF format. For all analyses, average mean values were calculated from eight randomly captured images per mouse.

For examination of CD45- and FOXP3-IR in cross sections, 16-bit images (field of view 0.4 mm^2^) were analyzed by binary thresholding. Identical threshold levels were set for every image. Binary particles were analyzed to obtain the total area (mm^2^) of CD45-IR per image^84^. This area was designated a region of interest and applied to binary threshold images of FOXP3-IR. The total area of co-localized CD45- and FOXP3-IR was measured (mm^2^). MAP-2-IR was assessed in colon cross sections by quantifying the number of neurons in eight nonadjacent sections in a 0.04 mm^2^ field of view per image using the cell counter plugin of ImageJ software. To measure 8-OHdG adducts in MAP-2-IR myenteric neurons, 16-bit images were analyzed with a 0.04 mm^2^ field of view per image. Regions of interest were set over MAP-2-IR neurons. Images of 8-OHdG immunofluorescence were converted to binary images by thresholding and binary particles were analyzed to obtain the percentage area of 8-OHdG-IR within MAP-2-IR neurons. For LMMP preparations, leukocytes (CD-45), neurons (MAP-2), and HMGB1-expressing cells were quantified in images with a 0.1 mm^2^ field of view using the cell counter plugin of ImageJ software. The size of each myenteric ganglion was quantified (µm^2^). The number of MAP-2-IR neurons within this area was enumerated (number of neurons per 0.01 mm^2^ area of ganglia). For analysis of O_2_^−^ in the myenteric plexus, regions of interest were set to determine MitoSOX fluorescence within the myenteric ganglia in randomly acquired images (0.1 mm^2^ field of view). The fluorescence intensity of O_2_^−^ in ganglia was measured as the mean grey value (mean fluorescence intensity) of the pixels within regions of interest. H&E-stained colon sections were imaged using an Olympus BX53 microscope (Olympus Imaging, Sydney, Australia).

### RNA extraction

The distal colon was collected from mice, snap-frozen in liquid nitrogen immediately after culling, and stored at − 80 °C until RNA was extracted. Tissues were homogenized in TRIzol® reagent (Thermo Fisher Scientific, Melbourne, Australia) with a metallic bead beater (TissueLyser LT, Qiagen, Melbourne, Australia) and RNA extraction was conducted per manufacturers’ protocols. RNA was additionally processed using RNA collection filters of the RNeasy Mini Kit (Qiagen) to improve purity. The concentration of RNA in each sample was quantified by a Qubit 1.0 fluorometer (Invitrogen, Thermofisher, Melbourne, Australia) according to the manufacturer’s protocol. Contaminates (such as phenol) were evaluated in RNA samples using a DeNovix DS-11 spectrophotometer (Gene Target Solutions, Sydney, Australia) and the quality of RNA was assessed using a 2100 Bioanalyzer (Agilent Technologies) microfluidics platform with the RNA 6000 Nano Kit (Agilent Technologies) according to the manufacturer’s recommended procedures.

### High-throughput RNA-sequencing of mRNA

Samples of RNA (n = 7/group) from C57BL/6 and *Winnie* mice, treated with either sham vehicle or BM-MSCs, were pooled into groups containing equal concentrations of RNA totaling at least 3 µg of RNA at 100 ng/µL in nuclease-free water. Samples were submitted to the Australian Genome Research Facility (AGRF, Melbourne, Australia) for polyA purification of mRNA from total RNA samples, RNA-Seq library preparation, and high-throughput sequencing using a 100 bp single-end read protocol on the Illumina HiSeq 2500 System. Base calling was performed using HiSeq Control Software (HCS) v2.2.68 and Real Time Analysis (RTA) v1.18.66.3. Sequencing data were generated using the Illumina bcl2fastq 2.20 pipeline. To map raw reads, the STAR v2.6.0c program was used to align reads to the mouse reference genome (GRCm38)^85^. The alignment file was sorted by coordinates using Sequence Alignment/Map(SAM) tools v1.8.0^86^. Read summarization of the raw counts per gene was determined using featureCounts v1.6.2 program of the software package subread^87^. Differentially expressed genes (DEGs) between groups were identified using the NOISeq-sim protocol of the R package NOIseq with a probability score > 0.8^88^.

#### Gene set enrichment analysis

Gene set enrichment analysis (GSEA) was performed with GSEA v4.1.0 [build 27] (Broad Institute) software on gene lists pre-ranked by log2FC × probability value. Gene sets for molecular functions (gene ontology) and oxidative stress-related terms were obtained from MSigDB. To develop gene sets for colon-specific cell types, counts data and associated metadata of single-nuclei RNA-seq of the colons from adult C57BL/6 mice were acquired from the Broad Institute single cell portal https://singlecell.broadinstitute.org/single_cell/study/SCP1038/^36^. Data were analyzed using *Seurat* and the original authors’ definitions of cell types. Prior to visualization, data were randomly subsampled for 1000 cells per cell type. Dimension reduction was performed by running the NormalizeData, ScaleData, FindVariableFeatures, RunPCA, FindNeighbors (dims = 1:20) and RunUMAP (dims = 1:20) commands. Heatmaps for genes of interest identified in the aforementioned analysis were visualized using the web-based tool *Morpheus* and presented as Z-score distributions (across samples).

#### Ulcerative colitis gene expression data

Gene expression data on the transcriptome of IBD patients were obtained from the National Centre for Biotechnology Information (NCBI) Gene Expression Omnibus (GEO) public data repository. Data obtained in the GEO is de-identifiable before submission. The expression profile of the inflamed intestine from IBD patients and of the healthy colon regions from uninflamed controls undergoing resection of non-obstructive colorectal adenocarcinoma was produced by high-throughput sequencing using Illumina HiSeq 2500 platform and uploaded by Peters et al.^89^ as reads per kilobase of transcript, per million mapped reads (RPKM). Similar to our methods, poly-A purified mRNA was used in this study from RNA isolated using TRIzol obtained from samples of snap-frozen 5 mm fragments of excised tissue. These data are accessible through GEO series accession number GSE83687 at https://www.ncbi.nlm.nih.gov/geo/. Gene expression of colon samples was used from this dataset, including male (n = 14) and female (n = 20) controls, male (n = 19) and female (n = 11) patients with UC. Previously reported markers for UC utilized for comparisons to our mouse dataset were obtained from Holgersen et al.^35^.

### Organotypic culture of myenteric ganglia

Organotypic preparations of the muscularis propria of the distal colon were prepared as previously described^26,90^. The organotypic muscle sheets were loosely pinned into 24-well cell culture plates modified to contain a silicon elastomer (Sylgard; Dow Corning, USA). Preparations were incubated (37 °C, 5% CO_2_) for 24 h in α-MEM supplemented with 100 U/mL penicillin/streptomycin, 1% glutaMAX and 5% (v/v) FBS (Gibco®, Life Technologies, Melbourne, Australia, for all), unless stated otherwise. Hyperoxic O_2_ tension, and the chemical H_2_O_2_, were utilized as oxidative stimuli in organotypic preparations. A hyperoxic environment was formed using a self-contained modular incubator (Billups-Rothenberg, Inc., San Diego, CA, USA) purged with a gas mixture of 95% O_2_ and 5% CO_2_. Organotypic cultures were exposed to H_2_O_2_ at a final concentration of 100 µM. Organotypic preparations were co-cultured with BM-MSCs at a concentration of 1 × 10^5^ cells per well. BM-MSCs were either directly cultured in the wells of organotypic tissues, or, for paracrine experiments, placed in a transwell semipermeable insert (0.4 µm pore size; Sigma-Aldrich) that contained the same media as organotypic cultures. The SOD1 antagonist LCS-1 (Sigma-Aldrich), was applied to organotypic cultures at a final concentration of 10 µM. For these experiments, the dimethyl sulfoxide (DMSO) vehicle for LCS-1 was present in all cultures at a concentration of 0.05% (v/v). Organotypic preparations were cultured for 24 h before being fixed overnight at 4 °C in Zamboni’s fixative (2% formaldehyde and 0.2% picric acid).

### Statistical analysis

Data analysis was performed using GraphPad Prism v7 (GraphPad Software Inc., San Diego, USA). A one-way ANOVA with a post hoc Holm-Sidak test was performed for multiple comparisons. Pearson correlation calculations were performed for X and Y values and slopes were determined using a linear regression analysis. For all analyses, *P* ≤ 0.05 was considered significant. All data were presented as mean ± standard error of the mean (SEM).

### Supplementary Information

Supplementary Figures.

Supplementary Tables.

## Data Availability

Sequencing data utilized in this study are made available as supplementary material (Data S1). Any other data generated in the current study are available from the corresponding author on request.

## References

[1] Cosnes J, Gower-Rousseau C, Seksik P (2011). Epidemiology and natural history of inflammatory bowel diseases. Gastroenterology.

[2] Bernell O, Lapidus A, Hellers G (2000). Risk factors for surgery and postoperative recurrence in Crohn’s disease [in eng]. Ann Surg.

[3] Schneider S, Wright CM, Heuckeroth RO (2019). Unexpected roles for the second brain: Enteric nervous system as master regulator of bowel function. Annu. Rev. Physiol..

[4] Furness JB (2012). The enteric nervous system and neurogastroenterology. Nat. Rev. Gastroenterol. Hepatol..

[5] De Giorgio R, Barbara G, Furness JB (2007). Novel therapeutic targets for enteric nervous system disorders. Trends Pharmacol. Sci..

[6] Lomax AE, Fernández E, Sharkey KA (2005). Plasticity of the enteric nervous system during intestinal inflammation. Neurogastroenterol. Motil..

[7] Lakhan SE, Kirchgessner A (2010). Neuroinflammation in inflammatory bowel disease. J. Neuroinflammation.

[8] Hansen MB (2003). The enteric nervous system III: A target for pharmacological treatment. Pharmacol. Toxicol..

[9] Poli E, Lazzaretti M, Grandi D (2001). Morphological and functional alterations of the myenteric plexus in rats with TNBS-induced colitis. Neurochem. Res..

[10] Krauter EM, Strong DS, Brooks EM (2007). Changes in colonic motility and the electrophysiological properties of myenteric neurons persist following recovery from trinitrobenzene sulfonic acid colitis in the guinea pig. Neurogastroenterol. Motil..

[11] Lomax AE, O'Hara JR, Hyland NP (2007). Persistent alterations to enteric neural signaling in the guinea pig colon following the resolution of colitis. Am. J. Physiol. Gastrointest. Liver Physiol..

[12] Bressenot A, Chevaux JB, Williet N (2013). Submucosal plexitis as a predictor of postoperative surgical recurrence in Crohn's disease. Inflamm. Bowel Dis..

[13] Ferrante M, de Hertogh G, Hlavaty T (2006). The value of myenteric plexitis to predict early postoperative Crohn's disease recurrence. Gastroenterology.

[14] Sokol H, Polin V, Lavergne-Slove A (2009). Plexitis as a predictive factor of early postoperative clinical recurrence in Crohn’s disease. Gut.

[15] Stavely R, Abalo R, Nurgali K (2020). Targeting enteric neurons and plexitis for the management of inflammatory bowel disease. Curr. Drug Targets.

[16] Bernardazzi, C,, Pêgo, B. & de Souza, H. S. P. Neuroimmunomodulation in the gut: Focus on inflammatory bowel disease. *Mediators Inflamm.* **2016** (2016). 10.1155/2016/1363818 PMC4947661 27471349

[17] Margolis KG, Gershon MD (2016). Enteric neuronal regulation of intestinal inflammation. Trends Neurosci..

[18] Forbes GM, Sturm MJ, Leong RW (2014). A phase 2 study of allogeneic mesenchymal stromal cells for luminal Crohn’s disease refractory to biologic therapy. Clin. Gastroenterol. Hepatol..

[19] Tian CM, Zhang Y, Yang MF (2023). Stem cell therapy in inflammatory bowel disease: A review of achievements and challenges. J. Inflamm. Res..

[20] Stavely R, Nurgali K (2020). The emerging antioxidant paradigm of mesenchymal stem cell therapy. Stem Cells Transl. Med..

[21] Kassis I, Vaknin-Dembinsky A, Karussis D (2011). Bone marrow mesenchymal stem cells: Agents of immunomodulation and neuroprotection. Curr. Stem Cell Res. Ther..

[22] Stavely R, Sakkal S, Stojanovska V (2014). Mesenchymal stem cells for the treatment of inflammatory bowel disease: From experimental models to clinical application. Inflamm. Regen..

[23] Gregoire C, Briquet A, Pirenne C (2018). Allogeneic mesenchymal stromal cells for refractory luminal Crohn’s disease: A phase I-II study. Dig. Liver Dis..

[24] Heazlewood CK, Cook MC, Eri R (2008). Aberrant mucin assembly in mice causes endoplasmic reticulum stress and spontaneous inflammation resembling ulcerative colitis. PLoS Med..

[25] Eri RD, Adams RJ, Tran TV (2011). An intestinal epithelial defect conferring ER stress results in inflammation involving both innate and adaptive immunity. Mucosal Immunol..

[26] Stavely R, Sahakian L, Filippone RT (2022). Oxidative stress-induced HMGB1 translocation in myenteric neurons contributes to neuropathy in colitis. Biomolecules.

[27] Stavely R, Robinson AM, Miller S (2015). Human adult stem cells derived from adipose tissue and bone marrow attenuate enteric neuropathy in the guinea-pig model of acute colitis. Stem Cell Res. Ther..

[28] Robinson AM, Miller S, Payne N (2015). Neuroprotective potential of mesenchymal stem cell-based therapy in acute stages of TNBS-induced colitis in guinea-pigs. PLoS ONE.

[29] Robinson AM, Rahman AA, Miller S (2017). The neuroprotective effects of human bone marrow mesenchymal stem cells are dose-dependent in TNBS colitis. Stem Cell Res. Ther..

[30] Robinson AM, Sakkal S, Park A (2014). Mesenchymal stem cells and conditioned medium avert enteric neuropathy and colon dysfunction in guinea pig TNBS-induced colitis. Am. J. Physiol. Gastrointest. Liver Physiol..

[31] Stavely R, Robinson AM, Miller S (2015). Allogeneic guinea pig mesenchymal stem cells ameliorate neurological changes in experimental colitis. Stem Cell Res. Ther..

[32] Wang M, Liang C, Hu H (2016). Intraperitoneal injection (IP), Intravenous injection (IV) or anal injection (AI)? Best way for mesenchymal stem cells transplantation for colitis. Sci. Rep..

[33] Forte D, Ciciarello M, Valerii MC (2015). Human cord blood-derived platelet lysate enhances the therapeutic activity of adipose-derived mesenchymal stromal cells isolated from Crohn's disease patients in a mouse model of colitis. Stem Cell Res. Ther..

[34] Cury DB, de Oliveira RA, Dalboni MA (2016). Comparative study of intravenous and topical administration of mesenchymal stem cells in experimental colitis. J. Transl. Sci..

[35] Holgersen K, Kutlu B, Fox B (2015). High-resolution gene expression profiling using RNA sequencing in patients with inflammatory bowel disease and in mouse models of colitis. J. Crohn's Colitis.

[36] Drokhlyansky E, Smillie CS, Van Wittenberghe N (2020). The human and mouse enteric nervous system at single-cell resolution. Cell.

[37] Misteli H, Koh CE, Wang LM (2015). Myenteric plexitis at the proximal resection margin is a predictive marker for surgical recurrence of ileocaecal Crohn's disease. Colorectal Dis..

[38] Nakao S, Itabashi M, Yamamoto T (2017). Predictive value of myenteric and submucosal plexitis for postoperative Crohn's disease recurrence. J. Anus Rectum Colon.

[39] Brown IAM, Gulbransen BD (2018). The antioxidant glutathione protects against enteric neuron death in situ, but its depletion is protective during colitis. Am. J. Physiol. Gastrointest. Liver Physiol..

[40] Klein D, Steens J, Wiesemann A (2017). Mesenchymal stem cell therapy protects lungs from radiation-induced endothelial cell loss by restoring superoxide dismutase 1 expression. Antioxid. Redox Signal..

[41] Sun Q, Wu W, Hu Y-C (2014). Early release of high-mobility group box 1 (HMGB1) from neurons in experimental subarachnoid hemorrhage in vivo and in vitro. J. Neuroinflammation.

[42] Janko C, Filipović M, Munoz LE (2014). Redox modulation of HMGB1-related signaling. Antioxid. Redox Signal..

[43] Tanaka F, Tominaga K, Ochi M (2008). Exogenous administration of mesenchymal stem cells ameliorates dextran sulfate sodium-induced colitis via anti-inflammatory action in damaged tissue in rats. Life Sci..

[44] Zuo D, Liu X, Shou Z (2013). Study on the interactions between transplanted bone marrow-derived mesenchymal stem cells and regulatory T cells for the treatment of experimental colitis. Int. J. Mol. Med..

[45] Gonzalez-Rey E, Anderson P, González MA (2009). Human adult stem cells derived from adipose tissue protect against experimental colitis and sepsis. Gut.

[46] Xie M, Qin H, Luo Q (2017). Comparison of adipose-derived and bone marrow mesenchymal stromal cells in a murine model of Crohn’s disease. Dig. Dis. Sci..

[47] Ando Y, Inaba M, Sakaguchi Y (2008). Subcutaneous adipose tissue–derived stem cells facilitate colonic mucosal recovery from 2, 4, 6-trinitrobenzene sulfonic acid (TNBS)–induced colitis in rats. Inflamm. Bowel Dis..

[48] Liang L, Dong C, Chen X (2011). Human umbilical cord mesenchymal stem cells ameliorate mice trinitrobenzene sulfonic acid (TNBS)-induced colitis. Cell Transplant..

[49] Zhang Q, Shi S, Liu Y (2009). Mesenchymal stem cells derived from human gingiva are capable of immunomodulatory functions and ameliorate inflammation-related tissue destruction in experimental colitis. J. Immunol..

[50] González MA, Gonzalez-Rey E, Rico L (2009). Adipose-derived mesenchymal stem cells alleviate experimental colitis by inhibiting inflammatory and autoimmune responses. Gastroenterology.

[51] Takeyama H, Mizushima T, Uemura M (2017). Adipose-derived stem cells ameliorate experimental murine colitis via TSP-1-dependent activation of latent TGF-β. Dig. Dis. Sci..

[52] Boyer L, Ghoreishi M, Templeman V (2005). Myenteric plexus injury and apoptosis in experimental colitis. Auton. Neurosci..

[53] Matteoli G, Gomez-Pinilla PJ, Nemethova A (2014). A distinct vagal anti-inflammatory pathway modulates intestinal muscularis resident macrophages independent of the spleen. Gut.

[54] Tsuchida Y, Hatao F, Fujisawa M (2011). Neuronal stimulation with 5-hydroxytryptamine 4 receptor induces anti-inflammatory actions via α7nACh receptors on muscularis macrophages associated with postoperative ileus. Gut.

[55] Thomas GAO, Rhodes J, Ingram JR (2005). Mechanisms of disease: Nicotine—A review of its actions in the context of gastrointestinal disease. Nat. Clin. Pract. Gastroenterol. Hepatol..

[56] McGrath J, McDonald JW, Macdonald JK (2004). Transdermal nicotine for induction of remission in ulcerative colitis. Cochrane Database Syst. Rev..

[57] Bernardini N, Segnani C, Ippolito C (2012). Immunohistochemical analysis of myenteric ganglia and interstitial cells of Cajal in ulcerative colitis. J. Cell Mol. Med..

[58] Nurgali K (2009). Plasticity and ambiguity of the electrophysiological phenotypes of enteric neurons. Neurogastroenterol. Motil..

[59] Nurgali K, Nguyen TV, Matsuyama H (2007). Phenotypic changes of morphologically identified guinea-pig myenteric neurons following intestinal inflammation. J. Physiol..

[60] Nurgali K, Nguyen TV, Thacker M (2009). Slow synaptic transmission in myenteric AH neurons from the inflamed guinea pig ileum. Am. J. Physiol. Gastrointest. Liver Physiol..

[61] Nurgali K, Qu Z, Hunne B (2011). Morphological and functional changes in guinea-pig neurons projecting to the ileal mucosa at early stages after inflammatory damage. J. Physiol..

[62] Oehmichen M, Reifferscheid P (1977). Intramural ganglion cell degeneration in inflammatory bowel disease. Digestion.

[63] Riemann J, Schmidt H (1982). Ultrastructural changes in the gut autonomic nervous system following laxative abuse and in other conditions. Scand. J. Gastroenterol. Suppl..

[64] Dora D, Ferenczi S, Stavely R (2021). Evidence of a myenteric plexus barrier and its macrophage-dependent degradation during murine colitis: Implications in enteric neuroinflammation. Cell. Mole. Gastroenterol. Hepatol..

[65] Piechota-Polanczyk A, Fichna J (2014). Review article: The role of oxidative stress in pathogenesis and treatment of inflammatory bowel diseases. Naunyn-Schmiedeberg's Arch. Pharmacol..

[66] Lih-Brody L, Powell SR, Collier KP (1996). Increased oxidative stress and decreased antioxidant defenses in mucosa of inflammatory bowel disease. Dig. Dis. Sci..

[67] Chandrasekharan B, Anitha M, Blatt R (2011). Colonic motor dysfunction in human diabetes is associated with enteric neuronal loss and increased oxidative stress. Neurogastroenterol. Motil. Off. J. Eur. Gastrointest. Motil. Soc..

[68] McQuade RM, Stojanovska V, Stavely R (2018). Oxaliplatin-induced enteric neuronal loss and intestinal dysfunction is prevented by co-treatment with BGP-15. Br. J. Pharmacol..

[69] McQuade RM, Carbone SE, Stojanovska V (2016). Role of oxidative stress in oxaliplatin-induced enteric neuropathy and colonic dysmotility in mice. Br. J. Pharmacol..

[70] Thrasivoulou C, Soubeyre V, Ridha H (2006). Reactive oxygen species, dietary restriction and neurotrophic factors in age-related loss of myenteric neurons. Aging Cell.

[71] Valle-Prieto A, Conget PA (2010). Human mesenchymal stem cells efficiently manage oxidative stress. Stem Cells Dev..

[72] Redondo J, Sarkar P, Kemp K (2018). Dysregulation of mesenchymal stromal cell antioxidant responses in progressive multiple sclerosis. Stem Cells Transl. Med..

[73] Sarkar P, Redondo J, Kemp K (2018). Reduced neuroprotective potential of the mesenchymal stromal cell secretome with ex vivo expansion, age and progressive multiple sclerosis. Cytotherapy.

[74] Musumeci D, Roviello GN, Montesarchio D (2014). An overview on HMGB1 inhibitors as potential therapeutic agents in HMGB1-related pathologies. Pharmacol. Ther..

[75] Huang L, Zhang D, Han W (2019). High-mobility group box-1 inhibition stabilizes intestinal permeability through tight junctions in experimental acute necrotizing pancreatitis. Inflamm. Res..

[76] Cheng X, Yang Y-L, Yang H (2018). Kaempferol alleviates LPS-induced neuroinflammation and BBB dysfunction in mice via inhibiting HMGB1 release and down-regulating TLR4/MyD88 pathway. Int. Immunopharmacol..

[77] Percie du Sert N, Hurst V, Ahluwalia A, Alam S, Avey MT, Baker M, Browne WJ, Clark A, Cuthill IC, Dirnagl U, Emerson M, Garner P, Holgate ST, Howells DW, Karp NA, Lazic SE, Lidster K, MacCallum CJ, Macleod M, Pearl EJ, Petersen O, Rawle F, Peynolds P, Rooney K, Sena ES, Silberberg SD, Steckler T, Wurbel H (2020). The ARRIVE guidelines 2.0: Updated guidelines for reporting animal research. PLoS Biol..

[78] Dominici M, Le Blanc K, Mueller I (2006). Minimal criteria for defining multipotent mesenchymal stromal cells. The International Society for Cellular Therapy position statement. Cytotherapy.

[79] Robinson AM, Stavely R, Miller S (2022). Mesenchymal stem cell treatment for enteric neuropathy in the Winnie mouse model of spontaneous chronic colitis. Cell Tissue Res..

[80] Stavely R, Fraser S, Sharma S (2018). The onset and progression of chronic colitis parallels increased mucosal serotonin release via enterochromaffin cell hyperplasia and downregulation of the serotonin reuptake transporter. Inflamm. Bowel Dis..

[81] Stavely R, Rahman AA, Sahakian L (2022). Divergent adaptations in autonomic nerve activity and neuroimmune signaling associated with the severity of inflammation in chronic colitis. Inflamm. Bowel Dis..

[82] Schneider CA, Rasband WS, Eliceiri KW (2012). NIH image to ImageJ: 25 years of image analysis. Nat. Methods.

[83] Rueden CT, Schindelin J, Hiner MC (2017). Image J2: ImageJ for the next generation of scientific image data. BMC Bioinform..

[84] Rahman AA, Robinson AM, Jovanovska V (2015). Alterations in the distal colon innervation in Winnie mouse model of spontaneous chronic colitis. Cell Tissue Res..

[85] Dobin A, Davis CA, Schlesinger F (2013). STAR: Ultrafast universal RNA-seq aligner. Bioinformatics.

[86] Li H, Handsaker B, Wysoker A (2009). The sequence alignment/map format and SAMtools. Bioinformatics.

[87] Liao Y, Smyth GK, Shi W (2014). featureCounts: An efficient general purpose program for assigning sequence reads to genomic features. Bioinformatics.

[88] Stavely R, Hotta R, Picard N (2022). Schwann cells in the subcutaneous adipose tissue have neurogenic potential and can be used for regenerative therapies. Sci. Transl. Med..

[89] Peters LA, Perrigoue J, Mortha A (2017). A functional genomics predictive network model identifies regulators of inflammatory bowel disease. Nat. Genet..

[90] Stavely R, Bhave S, Ho WLN (2021). Enteric mesenchymal cells support the growth of postnatal enteric neural stem cells. Stem Cells.

