# Supplementary material for: Bone marrow-derived mesenchymal stem cells mitigate chronic colitis and enteric neuropathy via anti-inflammatory and anti-oxidative mechanisms
Source: Sci Rep. 2024 Mar 20;14:6649. doi: 10.1038/s41598-024-57070-6 (PMC10951223; doi:10.1038/s41598-024-57070-6)
Supplement: Supplementary file 1 — Supplementary Figures. [file 41598_2024_57070_MOESM1_ESM.pdf]

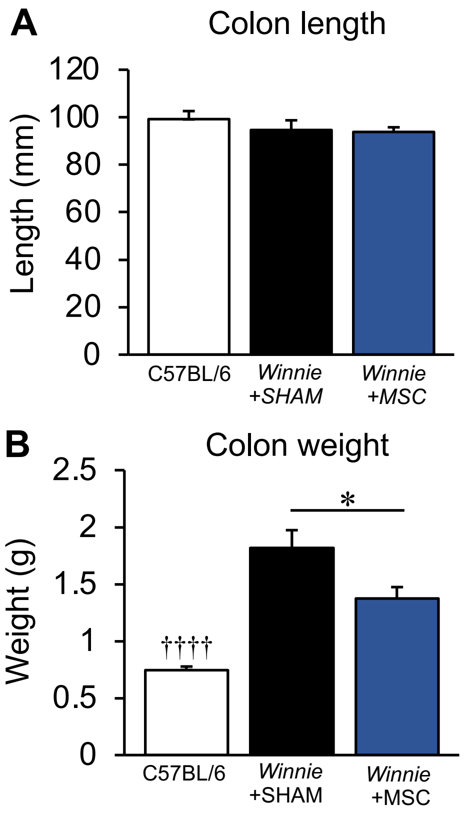

Supplementary Figure 1. ***Effects of BM-MSC treatments on the size of the colon in Winnie mice.*** Alterations in colon morphology measured by the length (mm) of the colon from caecum to rectum **(A)** and colon weight (g) **(B)** in C57BL/6 mice, *Winnie* mice treated with PBS (sham) and *Winnie* mice treated with BM-MSCs. **P*<0.05 between *Winnie*-sham and *Winnie*+MSC, ††††*P*<0.0001 between C57BL/6 and *Winnie*-sham: n=5 animals/group, *Winnie*+MSC: n=7 animals.

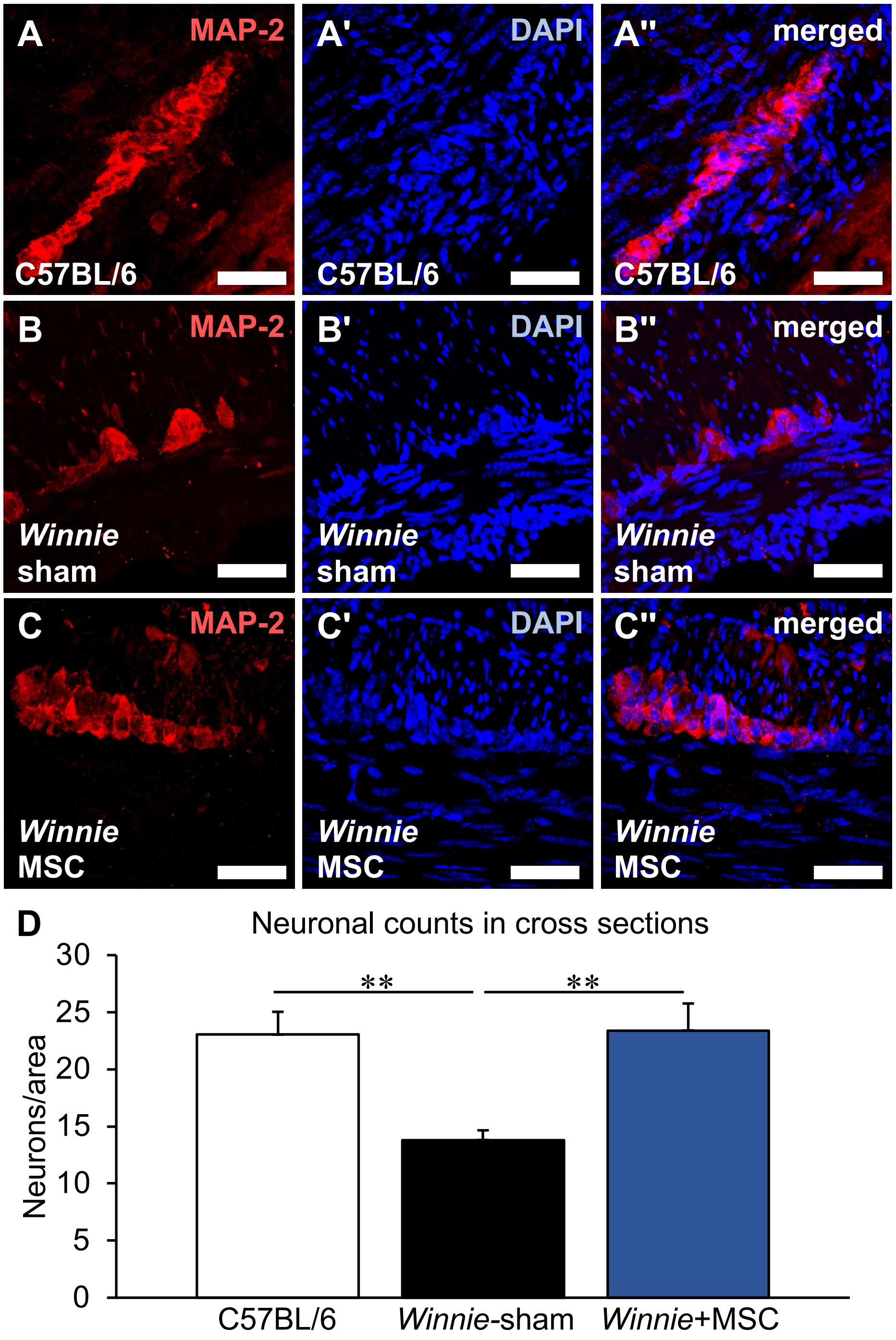

Supplementary Fig. 2. ***Effects of BM-MSC treatments on myenteric neurons in cross sections of distal colon from Winnie mice.*** **A-C″)** Neurons within the myenteric ganglia were observed by immunofluorescence using the neuronal marker MAP-2 **(A-C)** and the nuclear marker DAPI **(A′-C′)** in cross sections from the distal colon of C57BL/6 mice **(A-A″)**, sham-treated *Winnie* mice **(B-B″)** and *Winnie* mice treated with BM-MSCs **(C-C″)** (scale bar = 50µm). **D)** Quantification of myenteric neurons per area in colonic cross sections. ***P*<0.01; n=5 animals/group.

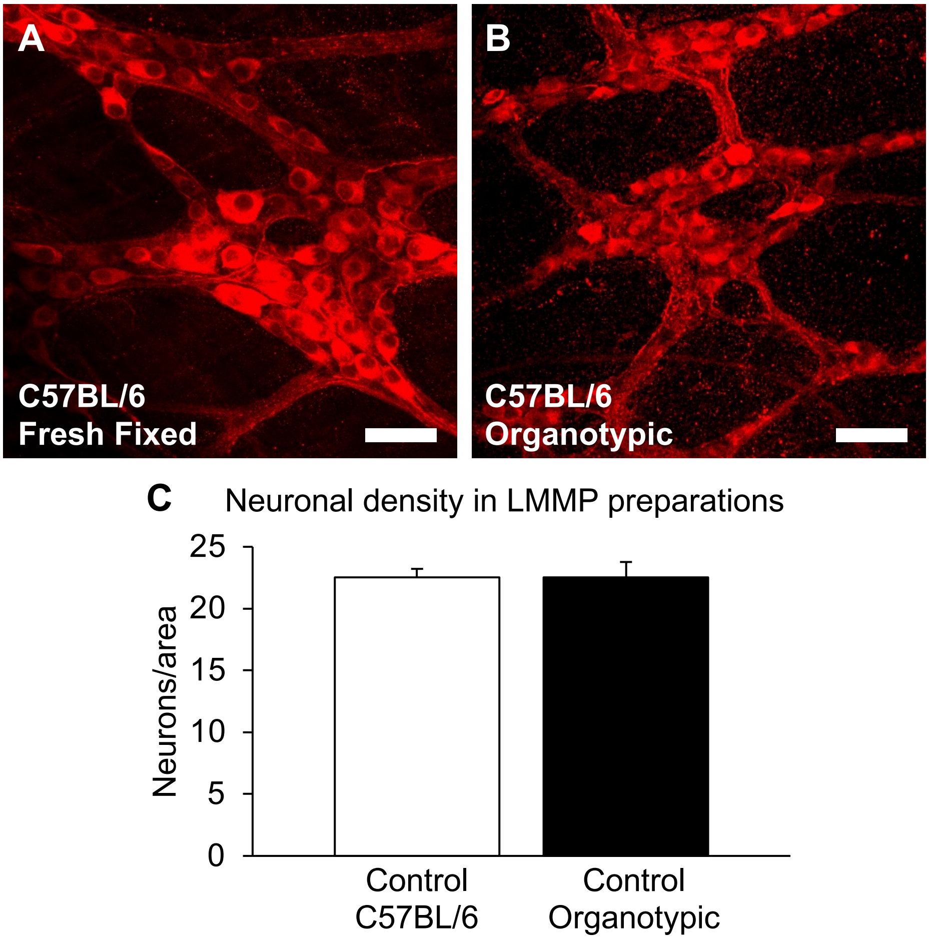

Supplementary Fig. 3. ***Evaluation of myenteric neuronal density in organotypic culture.*** **A-B)** Neurons within the myenteric ganglia were observed by immunofluorescence using the neuronal marker MAP-2 in wholemount LMMP preparations of the distal colon from C57BL/6 mice. Wholemounts that were fixed straight after the animal was culled **(A)** were compared to those subjected to organotypic culture for 24h **(B)** (scale bar = 50µm). **C)** Quantification of myenteric neuron density expressed as the number of neurons per ganglionated area; n=5 independent samples/group.

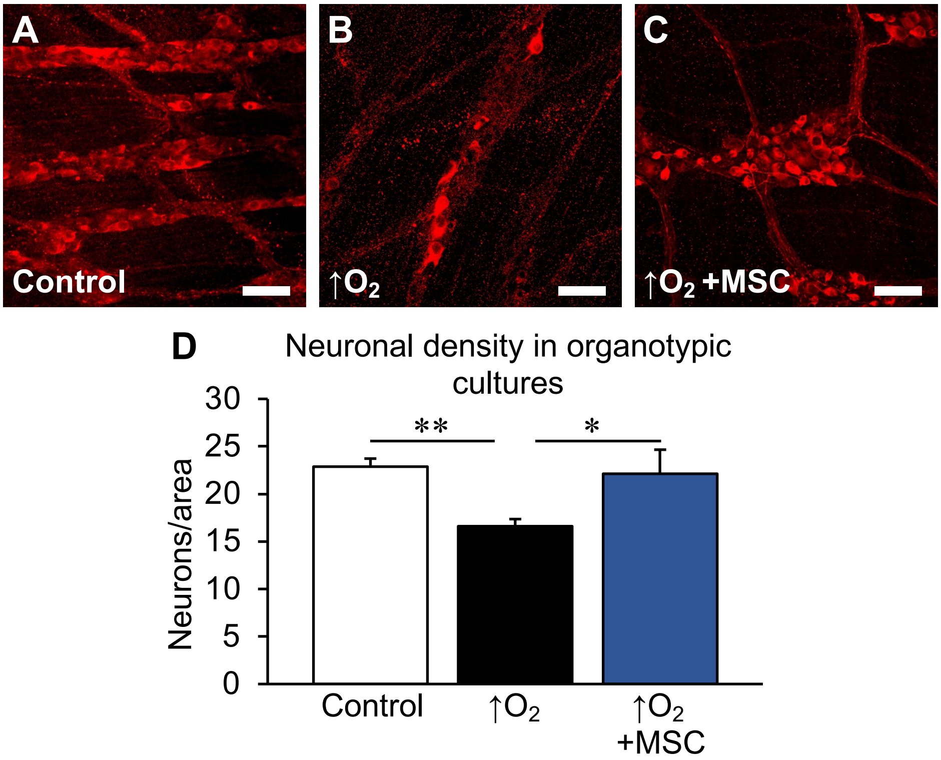

Supplementary Fig. 4. ***Effects of BM-MSCs on hyperoxia-induced myenteric neuropathy in organotypic culture.*** **A-C)** Neurons within the myenteric ganglia observed by immunofluorescence using the neuronal marker MAP-2 in distal colon organotypic cultures. Tissues were cultured for 24h in control **(A)**, hyperoxic (↑O_2) condition **(B)** and hyperoxia with $1x10^5$ BM-MSCs **(C)** (scale bar = 50µm). **D)** Quantification of myenteric neuron density expressed as the number of neurons per ganglionated area. **P*<0.05, ***P*<0.01; control: n=8 independent samples, ↑O_2: n=7 independent samples, ↑O_2 + MSC: n=5 independent samples.

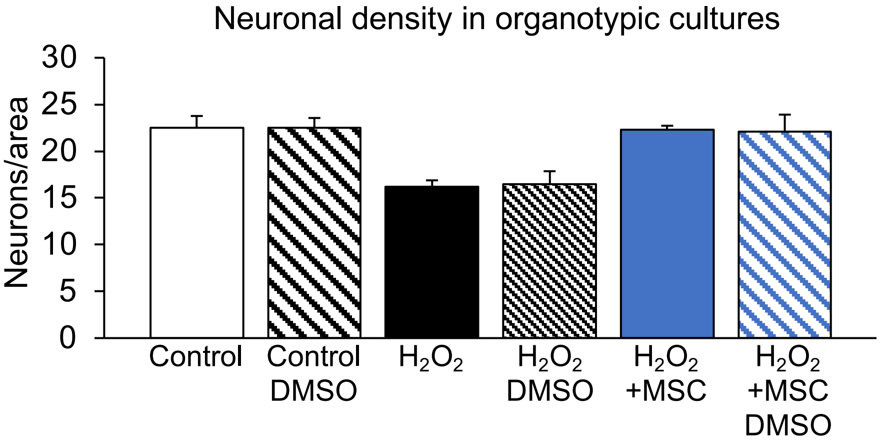

Supplementary Fig. 5. ***Effects of DMSO on organotypically-cultured myenteric neurons.*** Quantification of myenteric neuron density expressed as the number of neurons per ganglionated area. Myenteric neurons were quantified to test the effects of the solvent DMSO 0.05% (v/v) on MSC mediated neuroprotection. n=6 independent samples/group.
