# Supplementary material for: Bone marrow-derived mesenchymal stem cells mitigate chronic colitis and enteric neuropathy via anti-inflammatory and anti-oxidative mechanisms
Source: Sci Rep. 2024 Mar 20;14:6649. doi: 10.1038/s41598-024-57070-6 (PMC10951223; doi:10.1038/s41598-024-57070-6)
Supplement: Supplementary file 2 — Supplementary Tables. [file 41598_2024_57070_MOESM2_ESM.docx]

Table S1. GSEA using Gene ontology molecular functions database - *Winnie*-MSC vs *Winnie* sham

| **Description** | **Enrichment Score** | ***P* Value** | **Leading Edge Genes** | |
| --- | --- | --- | --- | --- |
| GOMF_CHEMOKINE_ACTIVITY | -0.8456712 | 0.0053286 | Cxcl1, Ccl19, Cxcl11, Ccl2, Cxcl10, Ccl11, Ccl17, Cxcl16, Cxcl9, Ccl8, Ccl7, Ccl5, Cxcl14, Ccl4, Ccl3, Cxcl5, Cxcl2, Cxcl3, Ppbp | |
| GOMF_CCR_CHEMOKINE_RECEPTOR_BINDING | -0.8435529 | 0.00943396 | Ccl25, Ccl20, Ccl22, Ccl19, Ccl2, Stat1, Ccl11, Ccl17, Ccl8, Ccrl2, Ccl7, Ccl5, Ccl4, Ccl3, Defb1 | |
| GOMF_CHEMOKINE_RECEPTOR_BINDING | -0.8347076 | 0.00512821 | Cxcl1, Ccl19, Cxcl11, Ccl2, Cxcl10, Stat1, Ccl11, Ccl17, S100a14, Cxcl16, Cxcl9, Ccl8, Ccrl2, Ccl7, Ccl5, Cxcl14, Ccl4, Ccl3, Cxcl5, Defb1, Cxcl2, Cxcl3, Ppbp | |
| GOMF_SERINE_HYDROLASE_ACTIVITY | -0.724842 | 0.001 | Klk15, Furin, Ctsk, Endou, Gzma, Mmp3, Plat, C2, Klk8, Prss12, Mmp7, Mmp9, F10, F3, Adam8, Mmp10, Mmp13, Tmprss11e, Hp, Mmp12, Mmp8, Prss22, Ltf, Klk11, Prss27, Klk14, Klk13, Klk7, Klk10, Tmprss11a, Tmprss11d | |
| GOMF_CYTOKINE_RECEPTOR_BINDING | -0.7095173 | 0.001 | Cxcl1, Tslp, Tnfsf10, Mif, Sdcbp, Fkbp1a, Ccl19, Cxcl11, Ccl2, Myd88, Bambi, Cxcl10, Il34, Casp8, Stat1, Vegfa, Ecm1, Ccl11, Eda, Il13ra1, Prlr, Ccl17, Il12rb1, Tnfsf9, S100a14, Cxcl16, Il6, Grem1, Cxcl9, Osmr, Ccl8, Il33, Ccrl2, Ccl7, Lif, Ccl5, Cxcl14, Pgf, Ccl4, Cd300lf, Ifng, Il1b, Tnf, Gata3, Lrg1, Ccl3, Cxcl5, Osm, Defb1, Cxcl2, Il1a, Cxcl3, Ppbp | |
| GOMF_CYTOKINE_ACTIVITY | -0.6934914 | 0.0027933 | Cxcl1, Tslp, Tnfsf10, Mif, Ccl19, Cxcl11, Ccl2, Nampt, Il17d, Inha, Fam3c, Cxcl10, Il34, Vegfa, Ccl11, Ccl17, Tnfsf9, Cxcl16, Il6, Timp1, Bmp8b, Grem1, Areg, Cxcl9, Ccl8, Il33, Ccl7, Thpo, Lif, Ccl5, Cxcl14, Inhba, Ccl4, Ifng, Il1b, Tnf, Ccl3, Cxcl5, Osm, Wnt7b, Cxcl2, Il1a, Tnfrsf11b, Spp1, Cxcl3, Ppbp | |
| GOMF_SIGNALING_RECEPTOR_REGULATOR_ACTIVITY | -0.6361712 | 0.001 | Mdk, Clec11a, Tnfsf13b, Egfr, Macc1, Cxcl1, Tslp, Tnfsf10, Mif, Hbegf, Ccl19, Cxcl11,Ccl2, Tgfa, Manf, Ly6g6d, Nampt, Il17d, Jag1, Sema6b, Inha, Fam3c, Adm2, Cxcl10, Il34, Vegfa, Ccl11, Eda, Nppc, Lep, Ly6e, Ccl17, Tnfsf9, Pthlh, Gpnmb, Igf1, Clec12a, Jag2, Cxcl16, Il6, Timp1, Bmp8b, Grem1, Endou, Areg, Penk, Cxcl9, Ccl8, Il33, Adh7, Pdyn, Retnlb, Lilrb4, Il18bp, Ccl7, Thpo, Lif, Ccl5, Cxcl14, Inhba, Cartpt, Fndc5, Fst, Pgf, Ccl4, Ifng, Cdc42ep2, Il1b, Adm, Tnf, Sema7a, Ccl3, Cxcl5, Osm, Calcb, Wnt7b, Cxcl2, Il1a, Tnfrsf11b, Spp1, Cxcl3, Ppbp, Pate4 | |
| GOMF_ENDOPEPTIDASE_ACTIVITY | -0.6296206 | 0.001 | Psmb9, Klk15, Furin, Ctsk, Psmb8, Gzma, Mmp3, Adamts12, Plat, Capns2, C2, Clca4, Sfrp1, Klk8, Mst1, Prss12, Mmp7, Usp18, Mmp9, F10, F3, Adam8, Clca2, Mmp10, Mmp13, Tmprss11e, Hp, Mmp12, Mmp8, Prss22, Ltf, Klk11, Prss27, Adam28, Klk14, Klk13, Klk7, Klk10, Asprv1, Tmprss11a, Tmprss11d | |
|  |  |  | |  |
|  |  |  | |  |

Table S2. GSEA using Gene ontology molecular functions database - *Winnie* sham vs C57BL/6

| **Description** | **Enrichment Score** | ***P* Value** | **Leading Edge Genes** |
| --- | --- | --- | --- |
| GOMF_CHEMOKINE_ACTIVITY | 0.9027785 | 0.001 | Cxcl9, Cxcl2, Ccl4, Cxcl10, Cxcl1, Cxcl3, Cxcl11, Ccl3, Ccl7, Cxcl5, Ccl2, Ccl5, Ccl8, Ccl20, Ccl22, Cxcl16, Cxcl14, Ccl17, Ppbp |
| GOMF_CCR_CHEMOKINE_RECEPTOR_BINDING | 0.83734995 | 0.001 | *Ccl4, Ccl3, Ccl7, Ccl2, Ccl5, Stat1, Ccl8, Ccl20, Ccl22, Ccrl2, Ccr2, Ccl17* |
| GOMF_CHEMOKINE_RECEPTOR_BINDING | 0.88322026 | 0.001 | *Cxcl9, Cxcl2, Ccl4, Cxcl10, Cxcl1, Cxcl3, Cxcl11, Ccl3, Ccl7, Cxcl5, Ccl2, Ccl5, Stat1, Ccl8, Ccl20, Ccl22, Cxcl16, Cxcl14, Ccrl2, S100a14, Ccr2, Ccl17, Ppbp* |
| GOMF_SERINE_HYDROLASE_ACTIVITY | 0.51991034 | 0.001 | *Mmp7, Mmp10, Mmp8, Mmp13, Hp, Gzma, Mmp12, Prss22, F10, Prss16, C2, Gzmb, Mmp3, Mmp9, Pcsk9, Prss27, Adam8, F3, Ctsc, Tmprss11a, Tmprss11d, Klk6, Prss12, Cfb* |
| GOMF_CYTOKINE_RECEPTOR_BINDING | 0.6373491 | 0.001 | *Il1rl1, Il18rap, Cd74, Cxcr2, Ccr5, Csf3r, Osmr, Il1r2, Il12b, Il18r1, Csf2rb, Ccr1, F3, Cxcr6, Il2ra, Ccrl2, Crlf2, Il5ra, Il12rb1, Ccr2, Ccr4, Il2rb, Il10ra, Cd4, Il7r, Ccr7, Ccr9, Il13ra1, Il3ra, Il21r, Cd44* |
| GOMF_CYTOKINE_ACTIVITY | 0.6551972 | 0.001 | Cxcl9, Cxcl2, Ifng, Ccl4, Il1b, Tnf, Cxcl10, Il1a, Osm, Tnfrsf11b, Cxcl1, Cxcl3, Spp1, Cxcl11, Ccl3, Ccl7, Cxcl5, Ccl2, Ccl5, Il11, Ccl8, Nrg1, Lif, Bmp8b, Tnfsf10, Timp1, Areg, Il12b, Ccl20, Fam3b, Ccl22, Thpo, Cxcl16, Cxcl14, Nampt, Inhba |
| GOMF_SIGNALING_RECEPTOR_  REGULATOR_ACTIVITY | 0.50252986 | 0.001 | Pate4, Cxcl9, Retnlb, Cxcl2, Ifng, Ccl4, Il1b, Tnf, Cxcl10, Il1a, Osm, Tnfrsf11b, Il18bp, Cxcl1, Cxcl3, Spp1, Cxcl11, Sema7a, Ccl3, Ccl7, Cxcl5, Ccl2, Ccl5, Il11, Ccl8, Lilrb4, Nrg1, Lif, Bmp8b, Tnfsf10, Timp1, Pcsk9, Areg, Clec12a, Il12b, Ccl20, Fam3b, Ccl22, Thpo, Cxcl16, Cxcl14, Insl6, Adm, Nampt, Ly6e, Manf, Inhba, Gmfg, Metrnl, Stc1, Tnfsf8, Stc2, Calcb, Ccl17, Ppbp, Il1rn, Ppy, Cdc42ep2, Hbegf, Il6, Cck, Tgfa, Nppc, Sema6b |

Table S3. Effects of mesenchymal stem cell treatments on endogenous factors in experimental colitis models

| Factor | Expression level in tissues after MSC treatment | References |
| --- | --- | --- |
| 15-LOX-1 | ↓Colon  ↓Spleen | (Mao et al., 2017)  (Mao et al., 2017) |
| Akt | ↑Colon | (Hoffman et al., 2018) |
| ARG1 | ↑Colon  ↑Serum | (de Aguiar et al., 2018, Song et al., 2018, Song et al., 2017c)  (Ferrer et al., 2016) |
| ARG2 | ↑Colon | (Sala et al., 2015) |
| bFGF | ↓Colon | (Tanaka et al., 2008) |
| BIP | ↓Colon | (Banerjee et al., 2015) |
| Ccl2 (MCP1) | ↓Colon  ↓Serum | (de Aguiar et al., 2018, Hoffman et al., 2018)  (Onishi et al., 2015) |
| CCL22 | ↑Colon | (Sala et al., 2015) |
| Ccl23 | ↑Colon | (Hoffman et al., 2018) |
| CCL5 | ↓Colon | (González et al., 2009, Gonzalez-Rey et al., 2009) |
| *CD11b* | ↓Colon | (Song et al., 2017c) |
| CXCL10 | ↓Colon  ↓Spleen | (Mao et al., 2017)  (Mao et al., 2017) |
| *Fizz1* | ↑Colon | (Song et al., 2018, Song et al., 2017c) |
| FOXP3 | ↑Colon    ↑Serum | (Sala et al., 2015, Wang et al., 2016, Jo et al., 2018, Nan et al., 2018, Martin Arranz et al., 2018, Chen et al., 2013)  (Sala et al., 2015) |
| Gata3 | ↑Colon | (Chen et al., 2013) |
| GSH | ↑Colon | (da Costa Gonçalves et al., 2017, Ferrer et al., 2016) |
| GSK3B | ↑Colon | (Hoffman et al., 2018) |
| HGF | ↓Colon | (Tanaka et al., 2008) |
| HO-1 | ↑Colon | (Sala et al., 2015) |
| IFN-γ | ↓Colon  N.D. Colon  ↓Serum  ↓CD4^+^ T lymphocytes in  spleen | (de Aguiar et al., 2018, Liu et al., 2015, Song et al., 2017c, Lin et al., 2015, Gonzalez-Rey et al., 2009, González et al., 2009, Liang et al., 2011, Chen et al., 2013, Wang et al., 2014, Zhang et al., 2009)  (Duijvestein et al., 2011)  (Forte et al., 2015, Chen et al., 2013)  (Wang et al., 2015)  (Ferrer et al., 2016) |
| IL-10 | ↑Colon  N.D. Colon  ↑Serum  ↓Serum  ↑MLN  ↑Spleen | (de Aguiar et al., 2018, Sala et al., 2015, Lee et al., 2016, Wang et al., 2016, Jo et al., 2018, Legaki et al., 2016, Song et al., 2018, Song et al., 2017c, Song et al., 2017b, Mao et al., 2017, Chao et al., 2016, Fu et al., 2018, Nan et al., 2018, González et al., 2009, Chen et al., 2013, Gonzalez-Rey et al., 2009, Zhang et al., 2009)  (Martin Arranz et al., 2018, Duijvestein et al., 2011)  (Heidari et al., 2018, Simovic Markovic et al., 2016)(Chen et al., 2013)  (Forte et al., 2015)  (Heidari et al., 2018)  (Heidari et al., 2018, Mao et al., 2017, Ferrer et al., 2016) |
| IL-12  (IL-12p40) | ↓Colon  ↓Serum | (Tang et al., 2015, Chao et al., 2016, González et al., 2009, Gonzalez-Rey et al., 2009)  (Nikolic et al., 2018, Ferrer et al., 2016) |
| IL-17 | ↓Colon  N.D. Colon  ↑Colon  ↓Serum  ↓MLN  ↓Spleen | (Liu et al., 2015, Song et al., 2017c, Song et al., 2017b, Fu et al., 2018, Nan et al., 2018, Liang et al., 2011, Chen et al., 2013, Wang et al., 2014, Zhang et al., 2009)  (Chao et al., 2016, Duijvestein et al., 2011)    (Hoffman et al., 2018)  (Heidari et al., 2018, Liang et al., 2011, Chen et al., 2013)  (Heidari et al., 2018)  (Heidari et al., 2018, Ferrer et al., 2016) |
| IL-1α | ↓Colon  ↓Serum | (Tang et al., 2015)  (Ferrer et al., 2016) |
| IL-1β | ↓Colon  ↓Serum  ↓Spleen | (Tang et al., 2015, Legaki et al., 2016, Hoffman et al., 2018, Song et al., 2017c, Song et al., 2017a, Lin et al., 2015, Mao et al., 2017, Onishi et al., 2015, González et al., 2009, Tanaka et al., 2008, He et al., 2012, Gonzalez-Rey et al., 2009)  (Simovic Markovic et al., 2016, Nikolic et al., 2018, Fu et al., 2018, González et al., 2009, Chen et al., 2013)  (Mao et al., 2017, Ferrer et al., 2016) |
| IL-2 | ↓Colon  ↓Serum  ↑CD4^+^ T lymphocytes in spleen | (Chen et al., 2013)  (Ferrer et al., 2016, Chen et al., 2013)  (Wang et al., 2015) |
| IL-21 | ↓Colon | (Chao et al., 2016) |
| IL-23 | ↓Colon | (Chao et al., 2016, Liang et al., 2011) |
| IL-4 | ↑Colon  ↑Serum | (Chen et al., 2013)  (Chen et al., 2013) |
| IL-5 | ↓Colon  ↓Serum | (Tang et al., 2015)  (Ferrer et al., 2016) |
| IL-6 | ↓Colon  ↓Serum  ↓Spleen | (Tang et al., 2015, de Aguiar et al., 2018, Liu et al., 2015, Hoffman et al., 2018, Song et al., 2018, Song et al., 2017a, Lin et al., 2015, Mao et al., 2017, Chao et al., 2016, Fu et al., 2018, González et al., 2009, Liang et al., 2011, Chen et al., 2013, Wang et al., 2014, Gonzalez-Rey et al., 2009, Zhang et al., 2009)  (Ferrer et al., 2016, Nikolic et al., 2018, Forte et al., 2015, González et al., 2009, Chen et al., 2013)  (Mao et al., 2017) |
| IL-8 | ↓Colon | (Ando et al., 2008) |
| MIF | ↓Colon | (Onishi et al., 2015) |
| MIP-2 | ↓Colon  N.D. Colon  ↓Serum | (González et al., 2009, Gonzalez-Rey et al., 2009)  (Onishi et al., 2015)  (González et al., 2009) |
| MMP2 | ↓Colon | (Legaki et al., 2016) |
| *Mrc1* (CD206) | ↑Colon | (Song et al., 2018, Song et al., 2017c) |
| *Nos2* (iNOS) | ↓Colon | (Song et al., 2018) |
| *P65/Rela* (NF-kbp65) | ↑Colon  ↓Colon | (Hoffman et al., 2018)  (Zuo et al., 2015) |
| PDI | ↓Colon | (Banerjee et al., 2015) |
| pSTAT3 | ↓Colon  ↓Spleen | (Jo et al., 2018)  (Mao et al., 2017) |
| RORyt | ↓Colon | (Nan et al., 2018, Chen et al., 2013) |
| SOD | ↑Colon | (Sun et al., 2015) |
| Tbx21 (T-Bet) | ↓Colon | (Chen et al., 2013) |
| TGFβ (1) | ↑Colon  N.D. Colon    ↑Serum  ↑MLN  ↑Spleen | (Heidari et al., 2018, Sala et al., 2015, Liu et al., 2015, Jo et al., 2018, Legaki et al., 2016, Song et al., 2017b) (Chao et al., 2016, Fu et al., 2018, Chen et al., 2013, Liu et al., 2015)  (Lee et al., 2016)  (Heidari et al., 2018, Simovic Markovic et al., 2016)  (Heidari et al., 2018)  (Heidari et al., 2018, Ferrer et al., 2016) |
| TNF-α | ↓Colon  N.D. Colon  ↓Serum  ↓Spleen | (Tang et al., 2015, de Aguiar et al., 2018, Liu et al., 2015, Wang et al., 2016, Legaki et al., 2016, Song et al., 2018, Song et al., 2017c, Lin et al., 2015, Mao et al., 2017, Chao et al., 2016, Onishi et al., 2015, Zuo et al., 2015, González et al., 2009, Tanaka et al., 2008, Chen et al., 2013, Wang et al., 2014, He et al., 2012, Gonzalez-Rey et al., 2009)  (Lee et al., 2016, Duijvestein et al., 2011)  (Simovic Markovic et al., 2016, Forte et al., 2015, Fu et al., 2018, Zuo et al., 2015, González et al., 2009, Liang et al., 2011, Chen et al., 2013, He et al., 2012)  (Mao et al., 2017, Ferrer et al., 2016) |
| TSG6 | ↑Colon  ↑Serum | (Sala et al., 2015, Wang et al., 2016)  (Sala et al., 2015, Fu et al., 2018) |
| VEGF | ↑Colon  ↓Colon | (Hoffman et al., 2018)  (Tanaka et al., 2008) |
| *Ym1* | ↑Colon | (Song et al., 2018, Song et al., 2017c) |

↑ increase, ↓ decrease, N.D. no difference; 15-LOX-1, 15-lipoxygenase-1; Akt, protein kinase B; ARG1, arginase 1; ARG2, arginase 2; bFGF, basic fibroblast growth factor; BIP, binding immunoglobulin protein; Ccl2 (MCP1), monocyte chemoattractant protein 1; CCL, C-C motif chemokine ligand; CD11b (ITGAM), integrin alpha M ; CXCL, C-X-C motif chemokine ligand; Fizz1, (Retnla), resistin-like alpha; FOXP3, forkhead box P3; Gata3, GATA binding protein 3; GSH, glutathione; GSK3B, glycogen synthase kinase 3 beta; HGF, hepatocyte growth factor; HO-1, haem oxygenase 1; IFN-γ, interferon gamma; IL, interleukin; MIF, macrophage migration inhibitory factor; MIP-2, macrophage inflammatory protein 2; MLN, mesenteric lymph nodes; MMP2, matrix metalloproteinase 2; Mrc1 (CD206), mannose receptor C-type 1; Nos2 (iNOS), inducible nitric oxide synthase; P65 (RELA), nuclear factor NF-kappa-B p65 subunit; PDI, protein disulphide isomerases; pSTAT3, phosphorylated signal transducer and activator of transcription 3; RORyt, RAR-related orphan receptor gamma; SOD, superoxide dismutase; Tbx21 (T-Bet), T-box protein expressed in T lymphocytes; TGFβ, transforming growth factor beta; TNF-α, tumour necrosis factor alpha; TSG6, tumour necrosis factor-inducible gene 6 protein; VEGF, vascular endothelial growth factor; Ym1 (Chil3), chitinase-like 3.

**Supplementary references**

ANDO, Y., INABA, M., SAKAGUCHI, Y., TSUDA, M., QUAN, G. K., OMAE, M., OKAZAKI, K. & IKEHARA, S. 2008. Subcutaneous adipose tissue–derived stem cells facilitate colonic mucosal recovery from 2, 4, 6‐trinitrobenzene sulfonic acid (TNBS)–induced colitis in rats. *Inflammatory Bowel Diseases,* 14**,** 826-838.

BANERJEE, A., BIZZARO, D., BURRA, P., DI LIDDO, R., PATHAK, S., ARCIDIACONO, D., CAPPON, A., BO, P., CONCONI, M. T., CRESCENZI, M., PINNA, C. M., PARNIGOTTO, P. P., ALISON, M. R., STURNIOLO, G. C., D'INCA, R. & RUSSO, F. P. 2015. Umbilical cord mesenchymal stem cells modulate dextran sulfate sodium induced acute colitis in immunodeficient mice. *Stem Cell Res Ther,* 6**,** 79.

CHAO, K., ZHANG, S., QIU, Y., CHEN, X., ZHANG, X., CAI, C., PENG, Y., MAO, R., PEVSNER-FISCHER, M., BEN-HORIN, S., ELINAV, E., ZENG, Z., CHEN, B., HE, Y., XIANG, A. P. & CHEN, M. 2016. Human umbilical cord-derived mesenchymal stem cells protect against experimental colitis via CD5(+) B regulatory cells. *Stem Cell Res Ther,* 7**,** 109.

CHEN, Q.-Q., YAN, L., WANG, C.-Z., WANG, W.-H., SHI, H., SU, B.-B., ZENG, Q.-H., DU, H.-T. & WAN, J. 2013. Mesenchymal stem cells alleviate TNBS-induced colitis by modulating inflammatory and autoimmune responses. *World Journal of Gastroenterology,* 19**,** 4702-4717.

DA COSTA GONÇALVES, F., GRINGS, M., NUNES, N. S., PINTO, F. O., GARCEZ, T. N. A., VISIOLI, F., LEIPNITZ, G. & PAZ, A. H. 2017. Antioxidant properties of mesenchymal stem cells against oxidative stress in a murine model of colitis. *Biotechnol Lett,* 39**,** 613-622.

DE AGUIAR, C. F., CASTOLDI, A., ANDRADE-OLIVEIRA, V., IGNACIO, A., DA CUNHA, F. F., FELIZARDO, R. J. F., BASSI, Ê. J., CÂMARA, N. O. S. & DE ALMEIDA, D. C. 2018. Mesenchymal stromal cells modulate gut inflammation in experimental colitis. *Inflammopharmacology,* 26**,** 251-260.

DUIJVESTEIN, M., WILDENBERG, M. E., WELLING, M. M., HENNINK, S., MOLENDIJK, I., VAN ZUYLEN, V. L., BOSSE, T., VOS, A. C. W., DE JONGE‐MULLER, E. S. & ROELOFS, H. 2011. Pretreatment with interferon‐γ enhances the therapeutic activity of mesenchymal stromal cells in animal models of colitis. *Stem Cells,* 29**,** 1549-1558.

FERRER, L., KIMBREL, E. A., LAM, A., FALK, E. B., ZEWE, C., JUOPPERI, T., LANZA, R. & HOFFMAN, A. 2016. Treatment of perianal fistulas with human embryonic stem cell-derived mesenchymal stem cells: a canine model of human fistulizing Crohn's disease. *Regen Med,* 11**,** 33-43.

FORTE, D., CICIARELLO, M., VALERII, M. C., DE FAZIO, L., CAVAZZA, E., GIORDANO, R., PARAZZI, V., LAZZARI, L., LAURETI, S., RIZZELLO, F., CAVO, M., CURTI, A., LEMOLI, R. M., SPISNI, E. & CATANI, L. 2015. Human cord blood-derived platelet lysate enhances the therapeutic activity of adipose-derived mesenchymal stromal cells isolated from Crohn's disease patients in a mouse model of colitis. *Stem Cell Res Ther,* 6**,** 170.

FU, Z. W., ZHANG, Z. Y. & GE, H. Y. 2018. Mesenteric injection of adipose-derived mesenchymal stem cells relieves experimentally-induced colitis in rats by regulating Th17/Treg cell balance. *Am J Transl Res,* 10**,** 54-66.

GONZÁLEZ, M. A., GONZALEZ–REY, E., RICO, L., BÜSCHER, D. & DELGADO, M. 2009. Adipose-derived mesenchymal stem cells alleviate experimental colitis by inhibiting inflammatory and autoimmune responses. *Gastroenterology,* 136**,** 978-989.

GONZALEZ-REY, E., ANDERSON, P., GONZÁLEZ, M. A., RICO, L., BÜSCHER, D. & DELGADO, M. 2009. Human adult stem cells derived from adipose tissue protect against experimental colitis and sepsis. *Gut,* 58**,** 929-939.

HE, X.-W., HE, X.-S., LIAN, L., WU, X.-J. & LAN, P. 2012. Systemic infusion of bone marrow-derived mesenchymal stem cells for treatment of experimental colitis in mice. *Digestive Diseases and Sciences,* 57**,** 3136-3144.

HEIDARI, M., POUYA, S., BAGHAEI, K., AGHDAEI, H. A., NAMAKI, S., ZALI, M. R. & HASHEMI, S. M. 2018. The immunomodulatory effects of adipose-derived mesenchymal stem cells and mesenchymal stem cells-conditioned medium in chronic colitis. *J Cell Physiol*.

HOFFMAN, J. M., SIDERI, A., RUIZ, J. J., STAVRAKIS, D., SHIH, D. Q., TURNER, J. R., POTHOULAKIS, C. & KARAGIANNIDES, I. 2018. Mesenteric Adipose-derived Stromal Cells From Crohn's Disease Patients Induce Protective Effects in Colonic Epithelial Cells and Mice With Colitis. *Cell Mol Gastroenterol Hepatol,* 6**,** 1-16.

JO, H., EOM, Y. W., KIM, H. S., PARK, H. J., KIM, H. M. & CHO, M. Y. 2018. Regulatory Dendritic Cells Induced by Mesenchymal Stem Cells Ameliorate Dextran Sodium Sulfate-Induced Chronic Colitis in Mice. *Gut Liver*.

LEE, H. J., OH, S. H., JANG, H. W., KWON, J. H., LEE, K. J., KIM, C. H., PARK, S. J., HONG, S. P., CHEON, J. H., KIM, T. I. & KIM, W. H. 2016. Long-Term Effects of Bone Marrow-Derived Mesenchymal Stem Cells in Dextran Sulfate Sodium-Induced Murine Chronic Colitis. *Gut Liver,* 10**,** 412-9.

LEGAKI, E., ROUBELAKIS, M. G., THEODOROPOULOS, G. E., LAZARIS, A., KOLLIA, A., KARAMANOLIS, G., MARINOS, E. & GAZOULI, M. 2016. Therapeutic Potential of Secreted Molecules Derived from Human Amniotic Fluid Mesenchymal Stem/Stroma Cells in a Mice Model of Colitis. *Stem Cell Reviews and Reports,* 12**,** 604-612.

LIANG, L., DONG, C., CHEN, X., FANG, Z., XU, J., LIU, M., ZHANG, X., GU, D. S., WANG, D. & DU, W. 2011. Human umbilical cord mesenchymal stem cells ameliorate mice trinitrobenzene sulfonic acid (TNBS)-induced colitis. *Cell Transplantation,* 20**,** 1395-1408.

LIN, Y., LIN, L., WANG, Q., JIN, Y., ZHANG, Y., CAO, Y. & ZHENG, C. 2015. Transplantation of human umbilical mesenchymal stem cells attenuates dextran sulfate sodium-induced colitis in mice. *Clin Exp Pharmacol Physiol,* 42**,** 76-86.

LIU, W., ZHANG, S., GU, S., SANG, L. & DAI, C. 2015. Mesenchymal Stem Cells Recruit Macrophages to Alleviate Experimental Colitis Through TGFβ1. *Cellular Physiology and Biochemistry,* 35**,** 858-865.

MAO, F., WU, Y., TANG, X., WANG, J., PAN, Z., ZHANG, P., ZHANG, B., YAN, Y., ZHANG, X., QIAN, H. & XU, W. 2017. Human umbilical cord mesenchymal stem cells alleviate inflammatory bowel disease through the regulation of 15-LOX-1 in macrophages. *Biotechnol Lett,* 39**,** 929-938.

MARTIN ARRANZ, E., MARTIN ARRANZ, M. D., ROBREDO, T., MANCHENO-CORVO, P., MENTA, R., ALVES, F. J., SUAREZ DE PARGA, J. M., MORA SANZ, P., DE LA ROSA, O., BUSCHER, D., LOMBARDO, E. & DE MIGUEL, F. 2018. Endoscopic submucosal injection of adipose-derived mesenchymal stem cells ameliorates TNBS-induced colitis in rats and prevents stenosis. *Stem Cell Res Ther,* 9**,** 95.

NAN, Z., FAN, H., TANG, Q., ZHANG, M., XU, M., CHEN, Q., LIU, Y., DONG, Y., WU, H. & DENG, S. 2018. Dual expression of CXCR4 and IL-35 enhances the therapeutic effects of BMSCs on TNBS-induced colitis in rats through expansion of Tregs and suppression of Th17cells. *Biochem Biophys Res Commun,* 499**,** 727-734.

NIKOLIC, A., SIMOVIC MARKOVIC, B., GAZDIC, M., RANDALL HARRELL, C., FELLABAUM, C., JOVICIC, N., DJONOV, V., ARSENIJEVIC, N., M, L. L., STOJKOVIC, M. & VOLAREVIC, V. 2018. Intraperitoneal administration of mesenchymal stem cells ameliorates acute dextran sulfate sodium-induced colitis by suppressing dendritic cells. *Biomed Pharmacother,* 100**,** 426-432.

ONISHI, R., OHNISHI, S., HIGASHI, R., WATARI, M., YAMAHARA, K., OKUBO, N., NAKAGAWA, K., KATSURADA, T., SUDA, G., NATSUIZAKA, M., TAKEDA, H. & SAKAMOTO, N. 2015. Human Amnion-Derived Mesenchymal Stem Cell Transplantation Ameliorates Dextran Sulfate Sodium-Induced Severe Colitis in Rats. *Cell Transplant,* 24**,** 2601-14.

SALA, E., GENUA, M., PETTI, L., ANSELMO, A., ARENA, V., CIBELLA, J., ZANOTTI, L., D’ALESSIO, S., SCALDAFERRI, F. & LUCA, G. 2015. Mesenchymal stem cells reduce colitis in mice via release of TSG6, independently of their localization to the intestine. *Gastroenterology,* 149**,** 163-176. e20.

SIMOVIC MARKOVIC, B., NIKOLIC, A., GAZDIC, M., NURKOVIC, J., DJORDJEVIC, I., ARSENIJEVIC, N., STOJKOVIC, M., LUKIC, M. L. & VOLAREVIC, V. 2016. Pharmacological Inhibition of Gal-3 in Mesenchymal Stem Cells Enhances Their Capacity to Promote Alternative Activation of Macrophages in Dextran Sulphate Sodium-Induced Colitis. *Stem Cells Int,* 2016**,** 2640746.

SONG, E. M., JUNG, S. A., LEE, K. E., JANG, J. Y., LEE, K. H., TAE, C. H., MOON, C. M., JOO, Y. H., KIM, S. E., JUNG, H. K. & SHIM, K. N. 2017a. The Therapeutic Efficacy of Tonsil-derived Mesenchymal Stem Cells in Dextran Sulfate Sodium-induced Acute Murine Colitis Model. *Korean J Gastroenterol,* 69**,** 119-128.

SONG, J. Y., KANG, H. J., HONG, J. S., KIM, C. J., SHIM, J. Y., LEE, C. W. & CHOI, J. 2017b. Umbilical cord-derived mesenchymal stem cell extracts reduce colitis in mice by re-polarizing intestinal macrophages. *Sci Rep,* 7**,** 9412.

SONG, W. J., LI, Q., RYU, M. O., AHN, J. O., BHANG, D. H., JUNG, Y. C. & YOUN, H. Y. 2018. TSG-6 released from intraperitoneally injected canine adipose tissue-derived mesenchymal stem cells ameliorate inflammatory bowel disease by inducing M2 macrophage switch in mice. *Stem Cell Res Ther,* 9**,** 91.

SONG, W. J., LI, Q., RYU, M. O., AHN, J. O., HA BHANG, D., CHAN JUNG, Y. & YOUN, H. Y. 2017c. TSG-6 Secreted by Human Adipose Tissue-derived Mesenchymal Stem Cells Ameliorates DSS-induced colitis by Inducing M2 Macrophage Polarization in Mice. *Sci Rep,* 7**,** 5187.

SUN, T., GAO, G. Z., LI, R. F., LI, X., LI, D. W., WU, S. S., YEO, A. E. & JIN, B. 2015. Bone marrow-derived mesenchymal stem cell transplantation ameliorates oxidative stress and restores intestinal mucosal permeability in chemically induced colitis in mice. *Am J Transl Res,* 7**,** 891-901.

TANAKA, F., TOMINAGA, K., OCHI, M., TANIGAWA, T., WATANABE, T., FUJIWARA, Y., OHTA, K., OSHITANI, N., HIGUCHI, K. & ARAKAWA, T. 2008. Exogenous administration of mesenchymal stem cells ameliorates dextran sulfate sodium-induced colitis via anti-inflammatory action in damaged tissue in rats. *Life Sciences,* 83**,** 771-779.

TANG, R. J., SHEN, S. N., ZHAO, X. Y., NIE, Y. Z., XU, Y. J., REN, J., LV, M. M., HOU, Y. Y. & WANG, T. T. 2015. Mesenchymal stem cells-regulated Treg cells suppress colitis-associated colorectal cancer. *Stem Cell Res Ther,* 6**,** 71.

WANG, C., CHEN, J., SUN, L. & LIU, Y. 2014. TGF-beta signaling-dependent alleviation of dextran sulfate sodium-induced colitis by mesenchymal stem cell transplantation. *Molecular Biology Reports,* 41**,** 4977-4983.

WANG, M., LIANG, C., HU, H., ZHOU, L., XU, B., WANG, X., HAN, Y., NIE, Y., JIA, S., LIANG, J. & WU, K. 2016. Intraperitoneal injection (IP), Intravenous injection (IV) or anal injection (AI)? Best way for mesenchymal stem cells transplantation for colitis. *Sci Rep,* 6**,** 30696.

WANG, W. Q., DONG, K., ZHOU, L., JIAO, G. H., ZHU, C. Z., LI, W. W., YU, G., WU, W. T., CHEN, S., SUN, Z. N., WANG, Y. M., LIU, W. T., ZHANG, J., WANG, B. M. & FENG, X. M. 2015. IL-37b gene transfer enhances the therapeutic efficacy of mesenchumal stromal cells in DSS-induced colitis mice. *Acta Pharmacol Sin,* 36**,** 1377-87.

ZHANG, Q., SHI, S., LIU, Y., UYANNE, J., SHI, Y., SHI, S. & LE, A. D. 2009. Mesenchymal stem cells derived from human gingiva are capable of immunomodulatory functions and ameliorate inflammation-related tissue destruction in experimental colitis. *The Journal of Immunology,* 183**,** 7787-7798.

ZUO, D., TANG, Q., FAN, H., SHOU, Z., LIU, X., CAO, D. & ZOU, Z. 2015. Modulation of nuclear factor-kappaB-mediated pro-inflammatory response is associated with exogenous administration of bone marrow-derived mesenchymal stem cells for treatment of experimental colitis. *Mol Med Rep,* 11**,** 2741-8.

|  | **Morphology** | **R square** | **F** | ***P* value** |
| --- | --- | --- | --- | --- |
| **Intra-ganglionic** | Round | 0.4688 | 11.47 | **0.0049** |
|  | Stellate | 0.06874 | 0.9596 | 0.3452 |
|  | Total | 0.4538 | 10.8 | **0.0059** |
| **Ganglia periphery** | Round | 0.7958 | 50.66 | **<0.0001** |
|  | Stellate | 0.7313 | 35.37 | **<0.0001** |
|  | Total | 0.8745 | 90.58 | **<0.0001** |
| **Extra-ganglionic** | Round | 0.4804 | 12.02 | **0.0042** |
|  | Stellate | 0.7898 | 48.84 | **<0.0001** |
|  | Total | 0.7475 | 38.49 | **<0.0001** |
| **All** | Round | 0.6699 | 26.38 | **0.0002** |
|  | Stellate | 0.7965 | 50.87 | **<0.0001** |
|  | Total | 0.8932 | 108.7 | **<0.0001** |

**Table S4. Linear regression correlations between leukocyte counts in proximity to the myenteric plexus and disease activity index scores**

Significant correlations in bold.

|  | **Morphology** | **R square** | **F** | ***P* value** |
| --- | --- | --- | --- | --- |
| **Intra-ganglionic** | Round | 0.3923 | 8.393 | **0.0125** |
|  | Stellate | 0.01053 | 0.138 | 0.7159 |
|  | Total | 0.4036 | 8.796 | **0.0109** |
| **Ganglia periphery** | Round | 0.4241 | 9.573 | **0.0085** |
|  | Stellate | 0.0767 | 1.08 | 0.3177 |
|  | Total | 0.2004 | 3.258 | 0.0943 |
| **Extra-ganglionic** | Round | 0.2361 | 4.019 | 0.0663 |
|  | Stellate | 0.08137 | 1.152 | 0.3027 |
|  | Total | 0.2147 | 3.555 | 0.0819 |
| **All** | Round | 0.3406 | 6.716 | **0.0224** |
|  | Stellate | 0.08187 | 1.159 | 0.3012 |
|  | Total | 0.2372 | 4.042 | 0.0656 |

Table S5. Linear regression correlations between leukocyte counts in proximity to the myenteric plexus and myenteric neuronal counts

Significant correlations in bold.
